# A systematic approach to the reporting of medically relevant findings from whole genome sequencing

**DOI:** 10.1186/s12881-014-0134-1

**Published:** 2014-12-14

**Authors:** Heather M McLaughlin, Ozge Ceyhan-Birsoy, Kurt D Christensen, Isaac S Kohane, Joel Krier, William J Lane, Denise Lautenbach, Matthew S Lebo, Kalotina Machini, Calum A MacRae, Danielle R Azzariti, Michael F Murray, Christine E Seidman, Jason L Vassy, Robert C Green, Heidi L Rehm, for The MedSeq Project

**Affiliations:** Harvard Medical School, Boston, Massachusetts USA; Laboratory for Molecular Medicine, Partners HealthCare Personalized Medicine, Cambridge, Massachusetts USA; Department of Pathology, Brigham and Women’s Hospital, Boston, Massachusetts USA; Divison of Genetics, Brigham and Women’s Hospital, Boston, Massachusetts USA; Cardiovascular Medicine, Brigham and Women’s Hospital, Boston, Massachusetts USA; Howard Hughes Medical Institute, Brigham and Women’s Hospital, Boston, Massachusetts USA; Department of Pediatrics, Boston Children’s Hospital, Boston, Massachusetts USA; Geisinger Health System, Danville, Pennsylvania USA; Broad Institute, Cambridge, Massachusetts USA; Section of General Internal Medicine, VA Boston HealthCare System, Boston, Massachusetts USA; Division of General Internal Medicine and Primary Care, Department of Medicine, Brigham and Women’s Hospital, Boston, Massachusetts USA

**Keywords:** Clinical genome sequencing, Incidental findings, MedSeq Project, Clinical report formatting

## Abstract

**Background:**

The MedSeq Project is a randomized clinical trial developing approaches to assess the impact of integrating genome sequencing into clinical medicine. To facilitate the return of results of potential medical relevance to physicians and patients participating in the MedSeq Project, we sought to develop a reporting approach for the effective communication of such findings.

**Methods:**

Genome sequencing was performed on the Illumina HiSeq platform. Variants were filtered, interpreted, and validated according to methods developed by the Laboratory for Molecular Medicine and consistent with current professional guidelines. The GeneInsight software suite, which is integrated with the Partners HealthCare electronic health record, was used for variant curation, report drafting, and delivery.

**Results:**

We developed a concise 5–6 page Genome Report (GR) featuring a single-page summary of results of potential medical relevance with additional pages containing structured variant, gene, and disease information along with supporting evidence for reported variants and brief descriptions of associated diseases and clinical implications. The GR is formatted to provide a succinct summary of genomic findings, enabling physicians to take appropriate steps for disease diagnosis, prevention, and management in their patients.

**Conclusions:**

Our experience highlights important considerations for the reporting of results of potential medical relevance and provides a framework for interpretation and reporting practices in clinical genome sequencing.

**Electronic supplementary material:**

The online version of this article (doi:10.1186/s12881-014-0134-1) contains supplementary material, which is available to authorized users.

## Background

Whole exome and genome sequencing, hereafter referred to as genomic sequencing (GS) are rapidly expanding into the clinical arena [1,2]. As the cost of GS declines and the performance and clinical utility of the technologies improve [3-6], it is likely that most clinical sequencing tests will be replaced by next generation sequencing of exomes and genomes in the near future, especially for indications with extensive genetic heterogeneity.

While the expansion of GS into clinical care is promising for the diagnosis and treatment of patients with genetic disorders, and eventually the screening of healthy individuals, GS produces an extensive amount of sequencing data which must be analyzed, filtered, interpreted, and reported upon by the clinical laboratory. In contrast to traditional genetic tests which typically report back a limited set of variants conditioned on prior clinical or family data and with relatively clearly defined supporting evidence, clinical reports resulting from GS analyses contain many variants each with disparate supporting evidence associated with a broad range of diseases, and a wide set of pretest probabilities. Physicians receiving GS reports must digest this complex information and determine the relevant inferences for the care of their patient. This requires a nuanced understanding of the underlying rationale for GS, technical aspects of the testing and its primary interpretation, and knowledge of how to define (or refine) the probabilistic nature of the genotype in the context of the individual patient or family. In many instances, this understanding is also dependent on mechanistic insight into the relevant human biology. Unfortunately, many physicians in non-genetic specialties lack fundamental genetic knowledge and are uncomfortable interpreting genetic test results, which may result in misinterpretation and inaccurate patient counseling [7-10]. Similarly, these non-specialists are often unaware of the phenotypic nuances and pathobiological knowledge necessary to optimize the interpretation of genotypes and realize the full potential of GS in clinical care. Together, these limitations increase the risk that physicians may over-interpret GS results, leading to unnecessary followup testing and/or inaccurate diagnoses; or under-interpret GS results, leading to inadequate patient management.

In order to facilitate the communication of results of potential medical relevance to a broad range of physicians, laboratories must decide upon the level of evidence required for variants to be returned on the report. Some laboratories may choose to report all classified variants, even variants of uncertain significance with little evidence supporting pathogenicity, resulting in massive reports. In this situation, physicians may be overwhelmed by lengthy reports and experience difficulty identifying which variants are most important with respect to the care of their patient. In contrast, laboratories may restrict reports to pathogenic variants with a definitive association with disease and a narrow definition of actionability. However few variants meet this level of evidence and this approach risks the omission of many variants whose relevance is dependent on the clinical context. Indeed, some variants may turn out to be clinically relevant as further clinical data are gathered. Thus, an intermediate approach may be required to facilitate the communication of results of potential medical relevance to a broad range of physicians. Moreover, it will be vital to communicate the requisite information on potential disease or risk associations for each reported variant to allow physicians to interpret GS test results in the relevant clinical context, to orchestrate additional phenotyping, or to refer the patient to an appropriate specialist.

To promote the understanding and utility of GS data, we have developed a Genome Report (GR) for the return of findings of potential medical relevance for individuals participating in the MedSeq Project, a randomized clinical trial assessing the impact of GS in two patient populations — healthy primary care patients and patients with cardiomyopathy of suspected genetic etiology. The GR is a concise 5–6 page genome-scale report featuring a succinct front page summary of findings of potential medical relevance with additional pages containing structured variant, gene, and disease information as well as supporting evidence and disease and health impact summaries.

## Methods

### MedSeq Project rationale and study design

The MedSeq Project is a randomized clinical trial that is testing approaches for evaluating and reporting of GS data and assessing the impact of integrating GS into primary care and cardiology settings. To achieve these goals, the MedSeq Project is recruiting 10 primary care physicians, each with 10 of their generally healthy patients and 10 cardiologists, each with 10 of their cardiomyopathy patients, for total of 200 participants. Half of the participants are being randomized to the GS arm. The physicians of each patient enrolled in the GS arm receive a GR communicating genetic findings of potential medical relevance. Interviews and survey instruments for both physicians and patients are being used to determine the impact of GS on attitudes, behaviors, healthcare utilization, and decision-making. A detailed report of the goals, protocol, and methods in the MedSeq Project is summarized elsewhere [11].

### Genome sequencing

GS is performed by the CLIA-certified, CAP-accredited Illumina Clinical Services Laboratory (San Diego, CA) using paired-end 100 base pair reads on the Illumina HiSeq platform [12]. Genomes are sequenced to at least 30X mean coverage and ≥ 95% of bases are sequenced to at least 8X coverage. Lossless BAM files containing sequence alignment and variant calling data are returned to the Laboratory for Molecular Medicine (LMM) via an encrypted portable hard drive for further analysis.

### Clinical bioinformatics pipeline

Lossless BAM files are converted to FASTQ format to obtain sequence read data and reads are realigned to the NCBI reference sequence (GRCh37) using the Burrows-Wheeler Aligner 0.6.1-r104 [13]. Variant calls are made using the Genomic Analysis Tool Kit (GATK) version 2.3-9-gdcdccbb [14] for all positions with ≥8X coverage. Variant annotation is derived from ALAMUT HT version 1.1.2, Variant Effect Predictor version 2.6 and the LMM’s GeneInsight laboratory database. Annotated variants are subsequently filtered to identify: (1) variants with a minor allele frequency (MAF) <5% in European American (EA) or African American (AA) chromosomes from the NHLBI Exome Sequencing Project (ESP; http://evs.gs.washington.edu/EVS/) classified as disease causing (DM) or possible disease causing mutations (DM?) in the Human Gene Mutation Database [15] or as Pathogenic or Likely pathogenic by the LMM; (2) nonsense, frameshift, and canonical splice-site (+/−1,2) variants with a MAF <1% in EA or AA chromosomes from the NHLBI ESP from a list of 4,631 disease-associated genes curated by expert review of many sources of gene-disease relationships (Online Mendelian Inheritance in Man (OMIM),ClinVar, etc.; http://www.iccg.org/iccg-member-toolbox/databases-tools/medial-exome-gene-list) and (3) pharmacogenomic variants for metformin (C11orf65 rs11212617), clopidogrel (CYP2C19 rs12248560, rs4244285, rs4986893, rs28399504, rs41291556, rs72552267, rs72558186, rs56337013), warfarin (CYP2C9 rs1057910, rs1799853, rs7900194, rs9332131, rs28371685, rs28371686 and VKORC1 rs9923231), simvastatin (SLCO1B1 rs4149056), and digoxin (ABCB1 rs1045642) metabolism. The disease-associated gene list is iteratively refined as gene-disease association information is curated. In addition, blood group antigens are predicted through a parallel pipeline as noted below.

### Variant classification

The principles we follow for the classification of potential Mendelian disease-causing variants have been previously described [16] and involve analysis of multiple lines of evidence including allele frequency, genetic and functional evidence from peer-reviewed scientific literature, and computational analysis (nucleotide and amino acid conservation, domain localization, missense pathogenicity prediction algorithms, and splice site prediction algorithms). Predicted loss-of-function (LOF) variants are evaluated in the context of review of known gene-disease associations and a determination of whether LOF is an established mechanism of disease for the gene in question. Each variant is classified according to American College of Medical Genetics and Genomics (ACMG) recommendations [17] and LMM criteria [16]. *Several variants required deliberation by MedSeq Project team members (including clinical geneticists, molecular geneticists, genetic counselors, and bioinformaticians) in order to assign a final classification.* The LMM uses a five tier classification system which includes the following classifications: benign, likely benign, uncertain significance, likely pathogenic, and pathogenic. In addition, a subset of uncertain significance variants are further subclassified into “uncertain significance: favor benign” or “uncertain significance: favor pathogenic”. With the exception of pharmacogenomic alleles and blood group antigens, only those variants with substantial evidence for causing or contributing to Mendelian genetic disease are reported. This includes all pathogenic, likely pathogenic, and uncertain significance: favor pathogenic variants. All reported disease-associated variants are confirmed via Sanger sequencing before reporting. Variants are fully reassessed when identified in a new case and the last assessment was completed over 1 year ago for pathogenic variants or over 6 months ago for likely pathogenic variants and variants of uncertain significance. Likely benign and benign variants are not fully reassessed unless requested by a clinician. However, all variants in the categories of likely pathogenic, uncertain significance, and likely benign are briefly reevaluated before each reporting through a quick search of online variant databases (ClinVar, HGMD, ESP, 1000G, ExAC) to ensure no new data has been reported.

### Pharmacogenomic analysis

A set of 18 variants associated with the metabolism of 5 drugs (see above) commonly used in the treatment of primary care and cardiology patients were selected for inclusion in the GR from PharmGKB Clinical Annotation Levels of Evidence Class I and Class II variants [18]. Additional PharmGKB Class I variants are also made available for validation and reporting if requested by the physician. All pharmacogenomic variant bases were genotyped using GATK version 2.3-9-gdcdccbb [14] and confirmed via Sanger sequencing or Illumina HumanOmni2.5 array (San Diego, CA) before reporting.

### Blood group prediction and serological confirmation

Red blood cell (RBC) and human platelet antigens (HPAs) are predicted using GS data. For all 45 RBC and 6 HPA genes, GATK is used to genotype each exon along with the first and last 10 bases of each intron. A custom prediction algorithm is then used to semi-automatically predict RBC and platelet antigens, followed by manual verification in the BAM alignment files for all antigens listed on the GR [19].

### Variant curation and reporting

Variant information, including classifications, interpretations, and associated references are stored in an internal laboratory knowledge base using the GeneInsight software suite [20]. Approved variant classifications with supporting evidence descriptions are submitted to the ClinVar database to support community knowledge sharing [21]. GRs are drafted and finalized using GeneInsight Lab and electronically delivered to physicians participating in the MedSeq Project via GeneInsight Clinic. Changes to variant classification in GeneInsight Lab resulting from new data are automatically communicated to participating physicians via an email notification that links them to a patient report update within the GeneInsight Clinic application for subsequent decision support [22].

### Development of the genome report

The GR presented herein was developed by a team of MedSeq Project investigators including physicians from genetic and non-genetic specialties, genetic counselors, molecular geneticists, and bioinformaticians. Prior to implementation with physicians and patients, the GR was evaluated by a multi-disciplinary advisory committee also consisting of physicians from genetic and non-genetic specialties, genetic counselors, molecular geneticists, bioinformaticians, bioethicists, patient advocates, and biotechnology industry leaders. Revised versions of the GR were subsequently reviewed with primary care physicians who were considering participation in MedSeq Project and underwent further changes intended to maximize clarity and utility (Additional file 1).

## Results

### Analysis and interpretation of genomes

**Figure 1:**
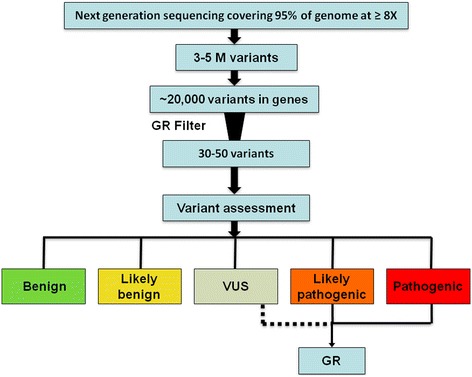
**MedSeq Project variant analysis workflow.** M (million), Genome Report (GR), variant of uncertain significance (VUS).

Each patient enrolled in the sequencing arm of the MedSeq Project receives a GR detailing findings of potential medical relevance including monogenic disease risk, carrier risk for recessive disorders, pharmacogenomic associations for commonly used medications, and a blood group antigen summary. The MedSeq Project has adopted an approach for returning findings of potential medical relevance that attempts to strike a balance between over and under reporting of variants. Instead of restricting reports to a very narrow set of disease-associated variants such as those found in the 56 genes recommended by the American College of Medical Genetics and Genomics (ACMG) [23], we have chosen to include additional analysis of many genes of potential medical relevance. This includes reporting of any variant with evidence supporting a causal role in a Mendelian genetic disease or representing carrier status for such diseases. The average genome-wide coverage ≥8X for the first 20 sequenced MedSeq Project cases (12 primary care arm and 8 cardiomyopathy arm) was 95.5% and patients had 3.4 to 5.3 million variants compared to the reference genome. Variants are filtered and analyzed via a comprehensive variant assessment process evaluating allele frequency, computational predictions, and genetic and functional evidence from peer-reviewed scientific literature (Figure 1) [16]. Each variant is classified according to LMM criteria [16], consistent with ACMG recommendations [17].

**Figure 2:**
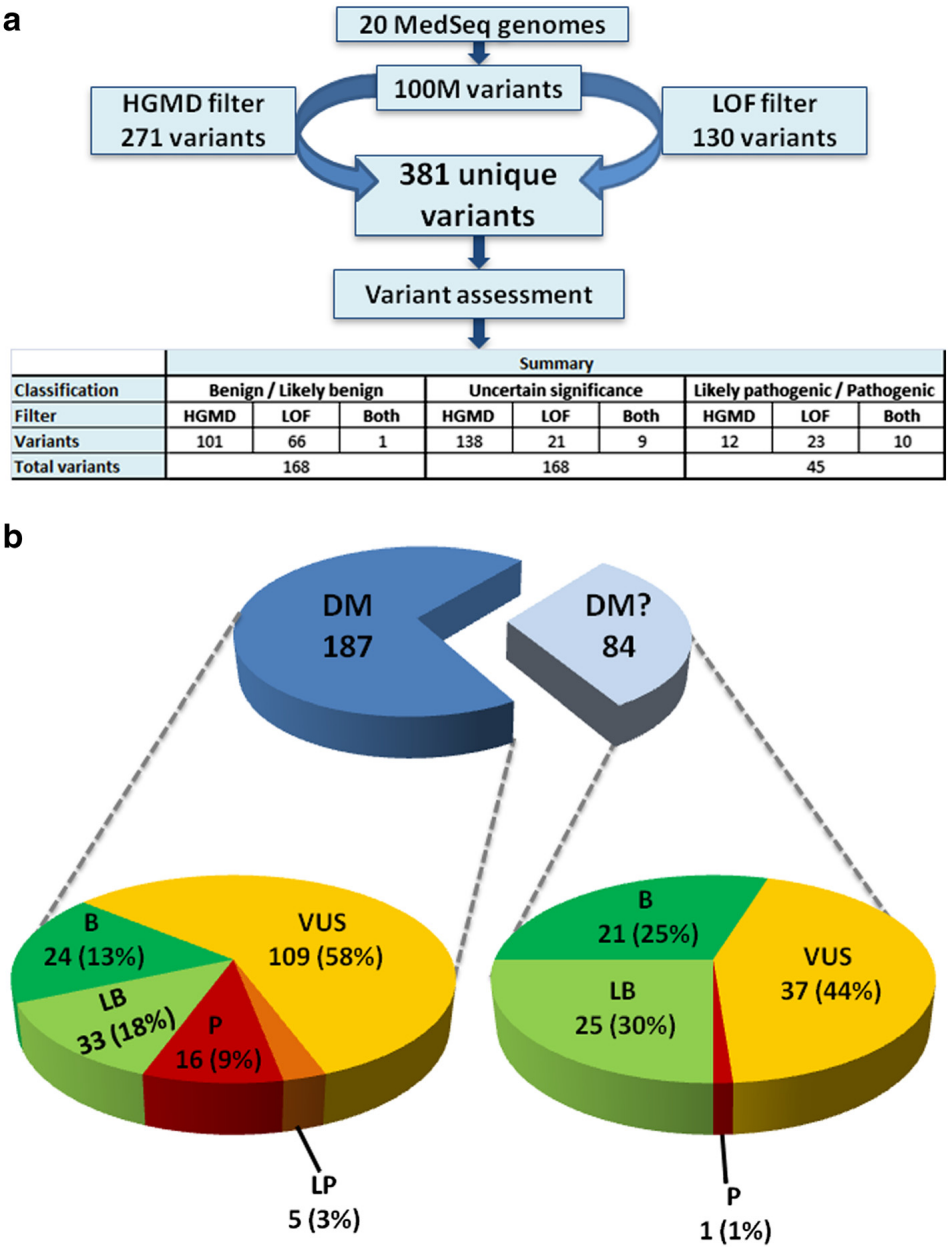
**Variant classifications from 20 genomes (a).** A total of 381 unique variants were assessed and classified after filtration **(b)** HGMD classification comparison. Disease causing mutation (DM), likely disease causing mutation (DM?), loss-of-function (LOF).

A total of 381 unique variants were manually assessed and classified for the first 20 MedSeq Project cases (251 ascertained via the HGMD filter, 110 ascertained via the LOF filter, 20 ascertained via both filters). After assessment, 168 (44%) of these variants were classified as benign or likely benign, 168 (44%) were classified as uncertain significance (including 5 classified as uncertain significance: favor pathogenic), and 45 (12%) were classified as likely pathogenic or pathogenic (Figure 2a). Similar to previously published studies [6,24,25], we found that very few variants with a purported disease association in HGMD met our criteria for pathogenicity. Out of 271 variants assessed that were categorized as DM or DM? in HGMD, only 22 (8%) were classified as likely pathogenic or pathogenic, and all but 1 of these had a DM categorization (Figure 2b).

A total of 80 unique variants from the first 20 MedSeq Project cases were selected for Sanger confirmation. Of these, 68 (85%) were confirmed, 1 (1%) was confirmed but with differing zygosity, and 11 (14%) were determined to be false positive. All false positive variants were either indels with low genotyping quality scores, or variants residing next to repetitive polynucleotide stretches. After final deliberation by the MedSeq Project team, 48/69 (69%) of the unique Sanger-confirmed variants were returned to the first 20 MedSeq Project participants (Table 1). These included 39 variants associated with carrier status for recessive disorders, 8 variants for monogenic disease risk (5 of which were identified in individuals from the cardiomyopathy arm and assumed to be responsible for these individual’s cardiomyopathy pending definitive analysis within the family) and 1 variant that conferred both carrier status and monogenic disease risk. The 21 variants that were Sanger confirmed but not reported were pursued for confirmation because the primary reviewer thought there was potential for being reported but after review by a board-certified laboratory geneticist and/or the full MedSeq Project committee, these variants did not meet the pathogenicity evidence level and/or clinical relevance threshold for return.

**Table 1:** **Reported findings of potential medical relevance for the first 20 MedSeq genomes**

**Gene**	**Nucleotide**	**Protein**	**Disease**	**Classification**	**Inheritance**	**Report section**	**Filter**
*SPATA7*	c.94 + 2 T > C	p.?	Leber congenital amaurosis	LP	AR	Carrier Risk	LOF
*ERCC5*	c.3238C > T	p.Arg1080X	Xeroderma pigmentosum	LP	AR	Carrier Risk	LOF
*COL7A1*	c.7557 + 1G > T	p.?	Epidermolysis bullosa dystrophica	LP	AR	Carrier Risk	LOF
*C2*	c.841_849 + 19del	p.Val281_Arg283del	C2 deficiency	LP	AR	Carrier Risk	LOF
*MYO7A*	c.5648G > A	p.Arg1883Gln	Usher syndrome type I	LP	AR	Carrier Risk	HGMD
*NAGA*	c.479C > G	p.Ser160Cys	Alpha-N-acetylgalactosaminidase deficiency	LP	AR	Carrier Risk	HGMD
*KCNQ1*	c.826delT	p.Ser276ProfsX13	Jervell and Lange-Nielsen syndrome	LP	AR	Carrier Risk	LOF
*LAMA2*	c.5563-2A > G	p.?	Congenital muscular dystrophy type IA	LP	AR	Carrier Risk	LOF
*SP110*	c.877A > T	p.Lys293X	Hepatic veno-occlusive disease with immunodeficiency	LP	AR	Carrier Risk	LOF
*ARSB*	c.1450A > G	p.Arg484Gly	Mucopolysaccharidosis type VI	LP	AR	Carrier Risk	HGMD
*BEST1*	c.602 T > C	p.Ile201Thr	Autosomal recessive bestrophinopathy	LP	AR	Carrier Risk	HGMD
*ACOX1*	c.1851delT	p.Gly618AlafsX24	Peroxisomal acyl-CoA oxidase deficiency	LP	AR	Carrier Risk	LOF
*LIFR*	c.2074C > T	p.Arg692X	Stuve-Wiedemann syndrome	LP	AR	Carrier Risk	LOF
*PAH*	c.842 + 5G > A	p.?	Phenylketonuria	LP	AR	Carrier Risk	HGMD, LOF
*MMACHC*	c.271dupA	p.Arg91LysfsX14	Methylmalonic aciduria and homocystinuria cblC type	P	AR	Carrier Risk	LOF
*CFTR*	c.3846G > A	p.Trp1282X	Cystic fibrosis	P	AR	Carrier Risk	HGMD, LOF
*PFKM*	c.237 + 1G > A	p.?	Glycogen storage disease 7	P	AR	Carrier Risk	HGMD, LOF
*CUBN*	c.6928_6934del	p.Glu2310CysfsX3	Imerslund-Gräsbeck syndrome	P	AR	Carrier Risk	LOF
*DUOX2*	c.3847 + 2 T > C	p.?	Hypothyroidism	P	AR	Carrier Risk	LOF
*ABCA4*	c.5882G > A	p.Gly1961Glu	Stargardt disease	P	AR	Carrier Risk	HGMD
*MPO*	c.2031-2A > C	p.?	Myeloperoxidase deficiency	P	AR	Carrier Risk	HGMD
*SERPINA1*	c.1096G > A	p.Glu366Lys	Chronic obstructive pulmonary disease	P	AR	Carrier Risk	HGMD
*USH2A*	c.1214del	p. Asn405IlefsX3	Usher syndrome type II	P	AR	Carrier Risk	LOF
*CLRN1*	c.528 T > G	p.Tyr176X	Usher syndrome type III	P	AR	Carrier Risk	HGMD, LOF
*CYP1B1*	c.171G > A	p.Trp57X	Primary congenital glaucoma	P	AR	Carrier Risk	LOF
*NLRP7*	c.337_338insG	p.Glu113GlyfsX7	Recurrent hydatidiform mole	P	AR	Carrier Risk	LOF
*BTD*	c.1330G > C	p.Asp444His	Biotinidase deficiency	P	AR	Carrier Risk	HGMD
*SPG7*	c.1529C > T	p.Ala510Val	Spastic paraplegia type 7	P	AR	Carrier Risk	HGMD
*PYGL*	c.25_44dup	p.Ser15ArgfsX21	Glycogen storage disease 6	P	AR	Carrier Risk	LOF
*WFS1*	c.124C > T	p.Arg42X	Wolfram syndrome	P	AR	Carrier Risk	LOF
*CYP1B1*	c.1103G > A	p.Arg368His	Primary congenital glaucoma	P	AR	Carrier Risk	HGMD
*TCIRG1*	c.1674-1G > A	p.?	Infantile malignant osteopetrosis	P	AR	Carrier Risk	HGMD, LOF
*LTBP4*	c.254delT	p.Leu85ArgfsX15	Cutis laxa, autosomal recessive, type IC	P	AR	Carrier Risk	LOF
*RAPSN*	c.264C > A	p.Asn88Lys	Congenital myasthenic syndrome	P	AR	Carrier Risk	HGMD
*TCTN2*	c.1877 T > A	p.Leu626X	Joubert syndrome	P	AR	Carrier Risk	LOF
*DUOX2*	c.2895_2898del	p.Phe966SerfsX29	Congenital hypothyroidism	P	AR	Carrier Risk	LOF
*HFE*	c.845G > A	p.Cys282Tyr	Hereditary hemochromatosis	P	AR	Carrier Risk	HGMD
*GJB2*	c.109G > A	p.Val37Ile	Hearing loss	P	AR	Carrier Risk	HGMD
*RAB27A*	c.259G > C	p.Ala87Pro	Familial hemophagocytic lymphohistiocytosis	VUS:FP	AR	Carrier Risk	HGMD
*CNGA3*	c.1669G > A	p.Gly557Arg	Achromatopsia	VUS:FP	AR	Carrier Risk	HGMD
*KCNQ1*	c.826delT	p.Ser276ProfsX13	Romano Ward syndrome	LP	AD	Monogenic	LOF
*MYBPC3*	c.3742-3759dup	p.Gly1248_Cys1253dup	Hypertrophic cardiomyopathy	LP	AD	Monogenic	HGMD
*MYBPC3*	c.2827C > T	p.Arg943X	Hypertrophic cardiomyopathy	P	AD	Monogenic	HGMD, LOF
*MYBPC3*	c.772G > A	p.Glu258Lys	Hypertrophic cardiomyopathy	P	AD	Monogenic	HGMD
*LHX4*	c.452-2A > C	p.?	Combined pituitary hormone deficiency	P	AD	Monogenic	LOF
*PTPN11*	c.1403C > T	p.Thr468Met	LEOPARD syndrome	P	AD	Monogenic	HGMD
*PPOX*	c.199delC	p.Leu67X	Variegate porphyria	P	AD	Monogenic	HGMD, LOF
*MYH7*	c.1987C > T	p.Arg663Cys	Hypertrophic cardiomyopathy	P	AD	Monogenic	HGMD
*ARSE*	c.410G > C	p.Gly137Ala	Chondrodysplasia punctata	VUS:FP	XL	Monogenic	HGMD

Uncertain significance: Favor pathogenic (VUS:FP), Likely pathogenic (LP), Pathogenic (P), autosomal dominant (AD), autosomal recessive (AR), X-linked (XL), loss-of-function (LOF), Human Gene Mutation Database (HGMD).

### An entire genome summarized on a single page

**Figure 3:**
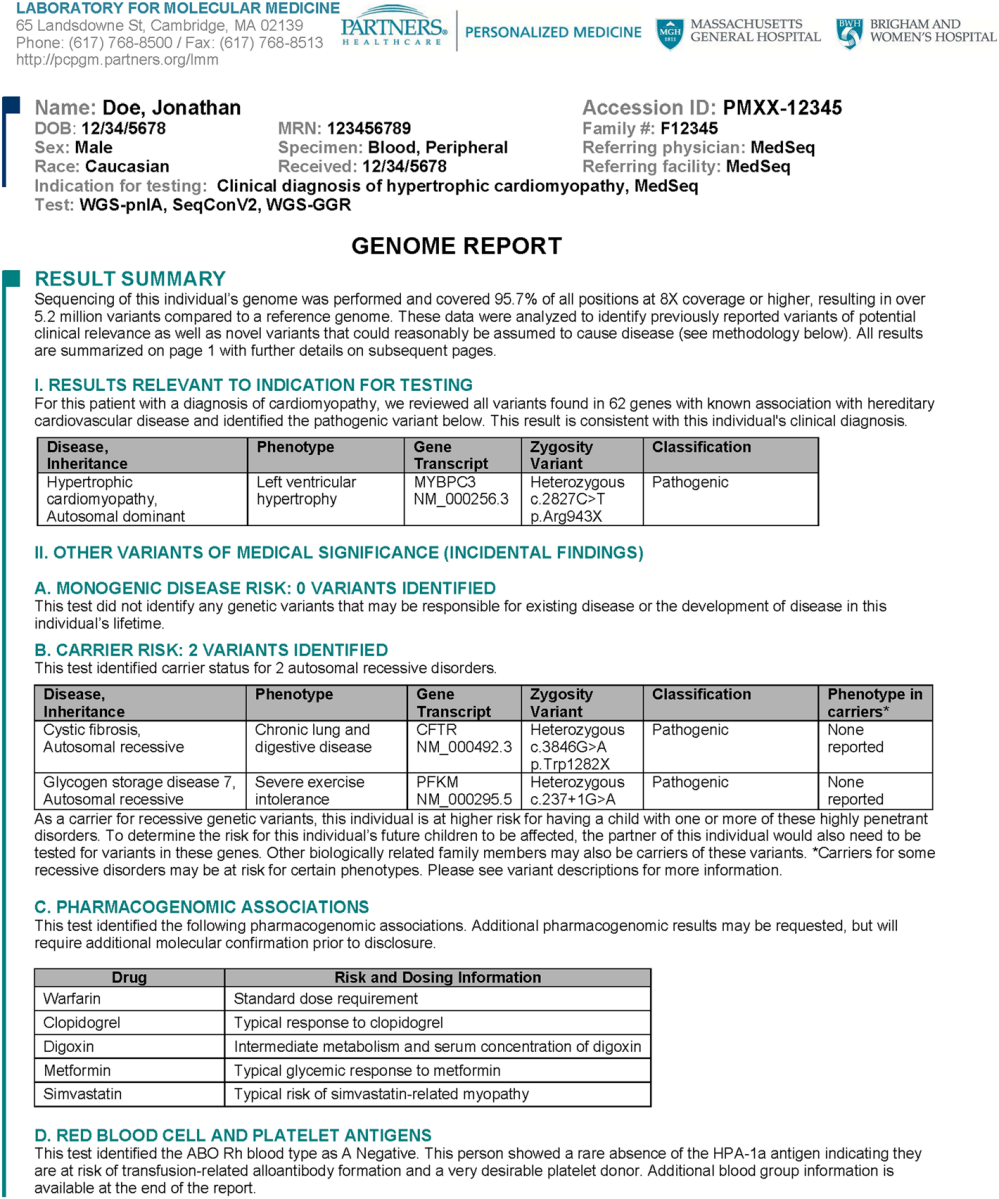
**Example Genome Report result summary.**

In order to communicate findings of potential medical relevance to MedSeq Project physicians, we developed a GR designed to convey complex genomic data in a succinct and effective manner to the non-genetic specialist physician (Figure 3 and Additional file 2, Additional file 3 and Additional file 4). The first page of the GR summarizes all findings including findings related to the indication for testing (for cardiomyopathy patients), monogenic disease risk, carrier risk for recessive disorders, pharmacogenomic results, and a blood group antigen summary on a single page (Figure 3). The GR result summary includes a description of genome coverage and the total number of variants identified compared to a reference genome, providing the physician with a high-level overview of the quality and complexity of their patient’s GS data. Results relevant to indication for testing (for cardiomyopathy patients) and other variants of medical significance (incidental findings) are clearly delineated and each is supplemented with the disease name, inheritance, a brief phenotype description, the Human Genome Organization (HUGO)-approved gene name, a variant description in Human Genome Variation Society (HGVS) nomenclature, and variant classification in a simple tabular format. When applicable, milder and/or low penetrant phenotypes that have been reported in association with carrier status for recessive disorders are also noted. For example, individuals who are carriers for pathogenic variants in the Wolfram syndrome 1 (*WFS1*) gene, may exhibit low frequency sensorineural hearing loss and/or diabetes mellitus [26-28]. A summary of pharmacogenomic associations for five commonly administered drugs are also displayed in a tabular format, describing predicted dose requirements, drug response, or the risk of adverse events in straightforward qualitative language. Finally, predicted ABO Rh blood types are returned along with any red blood cell (RBC) or human platelet antigens (HPAs) that are known to be rare in the population, enabling risk prediction for blood transfusion complications and/or awareness of desirable blood donor status for rare blood types. Physicians are directed to subsequent pages of the report to obtain more detailed information supporting the result summary.

### Supplementing reports with detailed variant, gene, and disease information

Due to the vast array of diseases, genes, and variant types that could be returned from GS, we sought to balance the need for a high level summary that enables quick viewing, with the importance of providing sufficient detail and evidence to support reported results. Therefore, we expand upon the single page GR summary with a detailed variant information section featuring structured tables with RefSeq transcript, variant frequency, disease prevalence, and if applicable, carrier frequency (Additional file 2, Additional file 3 and Additional file 4). Providing transcript information and a clear variant description is essential for an unequivocal definition of the variant. Reporting the population carrier frequency for recessive disorders, if known, allows physicians to counsel individuals on the risk of having an affected child with an existing or future reproductive partner. Variant interpretations are included for each variant describing the evidence collected during the variant assessment process and providing a rigorous rationale for the variant classification. These evidence-based variant summaries are then submitted to the ClinVar database to provide transparent rationales for variant interpretations. Disease information summaries outlining common phenotypes and natural history associated with the disease are extracted from GeneReviews and Orphanet abstracts [29,30]. Uniform resource locators (URLs) for relevant online resources are also provided, allowing physicians to consult appropriate materials. Familial risk information is also described to provide physicians and patients with an explanation of the inheritance pattern and potential risk implications for offspring and biological family members. Literature references are cited for each variant with a full list of references at the end of the report.

Pharmacogenomic results are supplemented with tabular supporting information (Additional file 2, Additional file 3 and Additional file 4). The drug and indication is provided along with a summary of the dose requirement or risk of adverse effects. For each drug, variants evaluated are described using HGVS nomenclature, rsID, and PharmGKB haplotype, when applicable. A detailed interpretation is provided for each drug and genotype frequencies are provided to allow physicians to contextualize the prevalence of the patient’s pharmacogenetic diplotype compared to the general population. Literature references are cited for each pharmacogenomic association with a full reference list at the end of the report.

The ABO Rh blood type and rare antigens summary are supplemented by additional blood group information provided in a tabular format (Additional file 2, Additional file 3 and Additional file 4). The presence or absence of a series of a selective number of 57 RBC antigens and all 33 platelet antigens are provided for each patient. The discussion also highlights when an individual is predicted to lack a highly prevalent antigen, making them at risk for transfusion-related events, when an individual lacks an antigen that may impact susceptibility to certain diseases, or when an individual lacks a highly immunogenic antigen, making them a good candidate for whole blood or platelet donation.

### Conveying methodology and limitations of genome sequencing

Laboratories offering GS employ a multitude of strategies for their bioinformatics, interpretation, and reporting pipelines [31,32]. In addition to providing required test methodology on the report, we also include a concise description of our analysis and interpretation process to promote understanding of the sequencing and interpretation pipeline, thereby allowing physicians to compare approaches among GS tests.

While our GS test currently offers at least 30X mean coverage across the genome and ≥ 95% of bases are sequenced to at least 8X coverage, there are some limitations to GS that the MedSeq Project team was tasked with conveying to physicians. First, certain types of variation are currently not reliably detected via GS including structural variants, triplet repeat expansions, copy number variants, uniparental disomy, and epigenetic changes. Therefore, the definitive absence of a pathogenic variant in certain disease-associated genes cannot always be reliably inferred depending on the spectrum of causative variation. Secondly, coverage of disease-associated genes may be insufficient to detect all variants. To address this limitation, we have opted to provide physicians with coverage information for any genes upon request and to include coverage information for established indication-associated genes for individuals from the cardiomyopathy arm of the study (Additional file 2 and Additional file 3). Finally, not all disease-associated genes have been identified and the clinical significance of variants in many genes, even those already associated with disease, remains elusive. The limitations described above are clearly listed on the first page and on the limitations section of each MedSeq Project GR.

### Delivery of genome reports

Our GeneInsight software suite [20] is used to store variant classifications, interpretations, diseases, and associated references for each reported variant. Reports are drafted using a custom GR reporting template and finalized reports are electronically delivered in portable document format (PDF) to physicians participating in the MedSeq Project via GeneInsight Clinic (GIC), a physician interface that facilitates report viewing and download as well as updates to reports over time [22]. Electronically transmitted reports also contain structured variant data in an XML packet that is represented in the patient’s electronic health record to enable clinical decision support in the future.

## Discussion

The reporting of findings of potential medical relevance from GS is rapidly expanding into the clinical arena. However, little attention has been focused on how to effectively communicate GS results to physicians. The MedSeq Project has adopted an experimental approach for the return of results of potential medical relevance to study the impact of introducing a variety of different categories of variant data into the clinical care setting. To convey this range of results to the MedSeq Project participants, we have created a physician-oriented genomics report featuring a concise single-page summary of genome-wide findings of potential medical relevance with clearly delineated sections for highly-penetrant monogenic disease risk, carrier status for recessive disorders, pharmacogenomic associations, and blood group antigens. Preliminary analysis of the first 18 audio-recorded GR disclosure sessions indicates that genetic and primary care physicians are generally able to synthesize essential report information and effectively communicate genetic disease risk to their patients [33-36].

The variant interpretation process required for clinical analysis of whole genome sequence is an arduous endeavor that begins with automated filtration, but always requires manual curation of publications and careful synthesis of available data. Members of the MedSeq Project team, including clinical geneticists, molecular geneticists, genetic counselors, and bioinformaticians, gather weekly to discuss variant evidence. We have found these discussions invaluable for reaching a consensus on the interpretation of difficult variants. Each genome analyzed thus far has contained pathogenic variants in genes and diseases unfamiliar to our clinical laboratory, further adding to the challenge of genomic interpretation. Our experience reinforces the notion that broad data sharing, including gene and variant interpretations, will be a prerequisite to effectively curate thousands of genes and the variation within them in order to improve the interpretation process and achieve consensus on the classification of variants. Community efforts supporting the deposition of variant data into centralized locations such as the ClinVar database [21] will be critical to the successful incorporation of GS into clinical medicine. The MedSeq Project will further support these efforts by depositing all generated variant classifications into the ClinVar database.

The GR reporting template presented here will continue to evolve as the clinical genomics community strives to promote the understanding and utility of GS data. Moving forward, we plan to incorporate hyperlinks to interfaces with supporting resources such as the Online Mendelian Inheritance in Man (OMIM), GeneReviews, PubMed, and disease-specific resources. Future iterations may include the addition of ancestry information and common disease risk alleles. In addition, the scope of genome analysis for each patient may evolve as an individual patient’s medical history and clinical situation develop. For example, additional pharmacogenomic results may be added and reinterpretation of genome data may be warranted if new symptoms arise or previously unappreciated familial risk is uncovered.

In the current genetic testing paradigm, clinical laboratories provide physicians with genetic results and physicians are in turn responsible for incorporating these results into care plans for their patients. One of the greatest barriers to integrating GS into clinical care is the limited amount of objective data regarding the best course of action for almost any genetic finding in the context of sequence from an entire genome. Physicians may grapple with ordering additional diagnostic tests for their patients to uncover potential disease-related phenotypes, and whether additional family members should be evaluated [2]. While this is not dissimilar to the problems encountered when any form of clinical testing moves from a narrow indication to broad clinical use, the sheer scale of the uncertainty is many orders of magnitude greater than for any prior scenario. In the future, it is clear that collaboration on a massive scale between clinical laboratories, clinical geneticists, genetic counselors, bioinformaticians, and physicians will be necessary to deconvolute the relationships between genotype and health or disease. A comprehensive redesign of the iterative process of test ordering, result reporting, and secondary interpretation will be necessary to provide integrated guidance and care recommendations for patients with genetic findings warranting further investigation. These steps will be critical as the results found in genome sequence reports will routinely cross the boundaries between existing silos of professional expertise.

## Conclusions

In order to advance the incorporation of GS data into clinical care, we have created a concise clinical GR that outlines findings of potential medical relevance, enabling physicians to counsel patients regarding the health and reproductive implications of their genome sequences. Our experience highlights important considerations in the reporting of findings of potential medical relevance and provides a framework for evolving interpretation and reporting practices in clinical GS.

## Additional files

Additional file 1:
**Development of the Genome Report.**
Additional file 2:
**Example Genome Report 1.** Cardiomyopathy patient with pathogenic variant. Additional file 3:
**Example Genome Report 2.** Cardiomyopathy patient with variant of uncertain significance. Additional file 4:
**Example Genome Report 3.** Primary care patient with risk allele.
