# Supplementary material for: A systematic approach to the reporting of medically relevant findings from whole genome sequencing
Source: BMC Med Genet. 2014 Dec 14;15:134. doi: 10.1186/s12881-014-0134-1 (PMC4342199; doi:10.1186/s12881-014-0134-1)
Supplement: Additional file 1: — Development of the Genome Report. [file 12881_2014_134_MOESM1_ESM.pdf]

Additional file 1. Development of the Genome Report

The GR has undergone iterative revisions and improvements during the study based upon early experiences in returning GR results and assessing physician understanding and feedback. Below are important issues and solutions raised during the development of the GR.

Result summary sections

There was considerable discussion surrounding how to combine versus separate diagnostic findings and secondary findings. Initially we had planned to have two separate reports; however, there are occasions when the distinction between incidental versus diagnostic findings is less clear, making separation difficult. In addition, having multiple reports can risk one getting lost. Therefore, we chose to focus on one report and began reporting all indication-specific and incidental findings in the Monogenic disease risk section of the report However, after receiving feedback from physicians and subsequent discussions amongst the MedSeq Project team, we ultimately decided to separate "Results Relevant to Indication for Testing" from "Other Variants of Medical Significance (Incidental Findings)" when the distinction is clear.

Variant inclusion

The MedSeq Project chose to report variants falling into pathogenic, likely pathogenic, and uncertain significance: favor pathogenic (VUS:FP) categories. This process differs from our routine clinical service offering which does not generally include variants in the VUS:FP category. Inclusion of VUS:FP variants in the MedSeq study was permitted given the controlled study environment and has allowed us to explore what happens when results of less certainty are returned to patients. In addition we felt that subsequent medical evaluation could aid in the determination of variant pathogenicity in some cases and our study had the resources to pursue certain additional studies. We chose to exclude benign, likely benign, uncertain significance: favor benign, and uncertain significance variants because we felt that including these variants would result in undesirably lengthy reports which may obscure variants of potential medical relevance. We also felt that absent the prior probability of family history or clinical features the reporting of variants falling below the classification of VUS:FP was unjustified.

Disease inclusion

We choose to go beyond the "minimum list" of 56 genes recommended by the American College of Medical Genetics and Genomics (ACMG) and expand our analysis to any gene previously associated with a Mendelian disease in order to have the highest yield of incidental findings with which to explore the aims of the study. In addition, we felt that systematic evaluation of genes claimed to be associated with Mendelian disease would allow ongoing improvement of the literature associated with gene-disease relationships that are used in diagnostic evaluations.

Blood group antigen inclusion

To our knowledge, GS data has not been previously used to determine blood group antigens for the purposes of clinical reporting. Including blood group antigens on MedSeq Project reports allowed us to assess the feasibility and utility of providing such information for determination of rejection risk from transfusions as well as determination of desirability for blood donation, a

critical clinical need in healthcare. Details of this effort and the validation of findings are being reported in an additional manuscript in preparation led by Dr. William Lane.

Gene-specific coverage metrics

In the initial version of the GR we choose to highlight coverage levels for any genes relevant to the indication for testing (*e.g.,* MedSeq patients from the cardiomyopathy cohort) that fell below 95% coverage. However, we later realized that additional information would be helpful to contextualize this information given that reduced coverage for a gene contributing 40% yield for HCM versus a gene contributing less than 1% yield should be considered differently. Therefore, each GR for cardiomyopathy cohort patients includes a table that lists the average coverage >8X for each gene with a known association with cardiomyopathy along with information regarding the relative contribution of each gene to the patient's specific type of cardiomyopathy (see Additional files 2-3). In addition, all patients may receive coverage information for any gene upon request.
