# Supplementary material for: A systematic approach to the reporting of medically relevant findings from whole genome sequencing
Source: BMC Med Genet. 2014 Dec 14;15:134. doi: 10.1186/s12881-014-0134-1 (PMC4342199; doi:10.1186/s12881-014-0134-1)
Supplement: Additional file 2: — Example Genome Report 1. Cardiomyopathy patient with pathogenic variant. [file 12881_2014_134_MOESM2_ESM.pdf]

LABORATORY FOR MOLECULAR MEDICINE
65 Landsdowne St, Cambridge, MA 02139
Phone: (617) 768-8500 / Fax: (617) 768-8513
http://pcpgm.partners.org/lmm

Name: Doe, Jonathan
Accession ID: PMXX-12345
DOB: 12/34/5678
MRN: 123456789
Family #: F12345
Sex: Male
Specimen: Blood, Peripheral
Referring physician: MedSeq
Race: Caucasian
Received: 12/34/5678
Referring facility: MedSeq
Indication for testing: Clinical diagnosis of hypertrophic cardiomyopathy, MedSeq
Test: WGS-pnlA, SeqConV2, WGS-GGR

GENOME REPORT

RESULT SUMMARY

Sequencing of this individual's genome was performed and covered 95.7% of all positions at 8X coverage or higher, resulting in over 5.2 million variants compared to a reference genome. These data were analyzed to identify previously reported variants of potential clinical relevance as well as novel variants that could reasonably be assumed to cause disease (see methodology below). All results are summarized on page 1 with further details on subsequent pages.

I. RESULTS RELEVANT TO INDICATION FOR TESTING

For this patient with a diagnosis of cardiomyopathy, we reviewed all variants found in 62 genes with known association with hereditary cardiovascular disease and identified the pathogenic variant below. This result is consistent with this individual's clinical diagnosis.

Disease, Inheritance	Phenotype	Gene Transcript	Zygosity Variant	Classification
Hypertrophic cardiomyopathy, Autosomal dominant	Left ventricular hypertrophy	MYBPC3 NM_000256.3	Heterozygous c.2827C>T p.Arg943X	Pathogenic

II. OTHER VARIANTS OF MEDICAL SIGNIFICANCE (INCIDENTAL FINDINGS)

A. MONOGENIC DISEASE RISK: 0 VARIANTS IDENTIFIED

This test did not identify any genetic variants that may be responsible for existing disease or the development of disease in this individual's lifetime.

B. CARRIER RISK: 2 VARIANTS IDENTIFIED

This test identified carrier status for 2 autosomal recessive disorders.

Disease, Inheritance	Phenotype	Gene Transcript	Zygosity Variant	Classification	Phenotype in carriers*
Cystic fibrosis, Autosomal recessive	Chronic lung and digestive disease	CFTR NM_000492.3	Heterozygous c.3846G>A p.Trp1282X	Pathogenic	None reported
Glycogen storage disease 7, Autosomal recessive	Severe exercise intolerance	PFKM NM_000295.5	Heterozygous c.237+1G>A	Pathogenic	None reported

As a carrier for recessive genetic variants, this individual is at higher risk for having a child with one or more of these highly penetrant disorders. To determine the risk for this individual's future children to be affected, the partner of this individual would also need to be tested for variants in these genes. Other biologically related family members may also be carriers of these variants. *Carriers for some recessive disorders may be at risk for certain phenotypes. Please see variant descriptions for more information.

C. PHARMACOGENOMIC ASSOCIATIONS

This test identified the following pharmacogenomic associations. Additional pharmacogenomic results may be requested, but will require additional molecular confirmation prior to disclosure.

Drug	Risk and Dosing Information
Warfarin	Standard dose requirement
Clopidogrel	Typical response to clopidogrel
Digoxin	Intermediate metabolism and serum concentration of digoxin
Metformin	Typical glycemic response to metformin
Simvastatin	Typical risk of simvastatin-related myopathy

D. RED BLOOD CELL AND PLATELET ANTIGENS

This test identified the ABO Rh blood type as A Negative. This person showed a rare absence of the HPA-1a antigen indicating they are at risk of transfusion-related alloantibody formation and a very desirable platelet donor. Additional blood group information is available at the end of the report.

GENOME REPORT (CONTINUED)

It should be noted that the disease risk section of this report is limited only to variants with strong evidence for causing highly penetrant disease, or contributing to highly penetrant disease in a recessive manner. Not all variants identified have been analyzed, and not all regions of the genome have been adequately sequenced. These results should be interpreted in the context of the patient's medical evaluation, family history, and racial/ethnic background. Please note that variant classification and/or interpretation may change over time if more information becomes available. For questions about this report, please contact the Genome Resource Center at GRC@partners.org

DETAILED VARIANT INFORMATION

I. RESULTS RELEVANT TO CLINICAL INDICATION

Disease<br>Inheritance	Gene<br>Transcript	Zygosity<br>Variant<br>Classification	Variant Frequency	Disease<br>Prevalence	References
Hypertrophic cardiomyopathy<br>Autosomal dominant	MYBPC3<br>NM_000256.3	Heterozygous<br>c.2827C>T<br>p.Arg943X<br>Pathogenic	Not previously reported	1/500	Van Driest 2004,<br>Lekanne Deprez 2006,<br>Tajsharghi 2010

VARIANT INTERPRETATION: The Arg943X variant in MYBPC3 has been reported in 8 individuals with HCM (Van Driest 2004, Lekanne Deprez 2006, Tajsharghi 2010, LMM unpublished data). Several of these individuals carried an additional clinically significant variant and presented with early onset disease (Lekanne Deprez 2006, Tajsharghi 2010, LMM unpublished data).
This variant leads to a premature stop at codon 943, which was shown to result in a stable mRNA, and is therefore predicted to generate a truncated protein (Lekanne Deprez 2006). Pathogenic nonsense variants in MYBPC3 are prevalent among individuals with HCM. In summary, this variant meets our criteria to be classified as pathogenic (http://pcpgm.partners.org/LMM).

DISEASE INFORMATION: Hypertrophic cardiomyopathy (HCM), caused by mutations in genes encoding components of the sarcomere, is characterized by left ventricular hypertrophy (LVH) in the absence of predisposing or existing cardiac conditions (e.g., aortic stenosis or long-standing hypertension). The clinical manifestations of HCM range from asymptomatic to progressive heart failure to sudden cardiac death. Common symptoms include shortness of breath, chest pain, palpitations, orthostasis, presyncope, and syncope. Adapted from GeneReviews: http://www.ncbi.nlm.nih.gov/books/NBK1768/

FAMILIAL RISK: HCM due to pathogenic variants in the MYBPC3 gene is typically inherited in an autosomal dominant pattern. Each first-degree relative has a 50% chance of inheriting the variant and its risk for disease.

II. OTHER VARIANTS OF MEDICAL SIGNIFICANCE (INCIDENTAL FINDINGS)

A. MONOGENIC DISEASE RISK

This test did not identify any genetic variants that may be responsible for existing disease or the development of disease in this individual's lifetime.

B. CARRIER RISK

Disease<br>Inheritance	Gene<br>Transcript	Zygosity<br>Variant<br>Classification	Variant<br>Frequency	Disease<br>Prevalence<br>(Carrier Freq.)	References	Phenotype in carriers
Cystic fibrosis<br>Autosomal recessive	CFTR<br>NM_000492.3	Heterozygous<br>c.3846G>A<br>p.Trp1282X<br>Pathogenic	6/8600<br>(0.07%)<br>European<br>American	1/3200<br>European<br>American<br>(1/25)	Hamosh 1991<br>Kerem 1990<br>Shoshani 1992<br>Vidaud 1990	None reported

VARIANT INTERPRETATION: The Trp1282X variant in CFTR has been identified in numerous patients with cystic fibrosis (Viduad 1990, Kerem1990, Hamosh 1991, Shoshani 1992). This variant is present on the American Board of Medical Genetics CFTR mutation panel (http://www.acmg.net/Pages/ACMG_Activities/stds-2002/cf.htm). This nonsense variant leads to a premature termination codon at position 1282, which is predicted to lead to a truncated or absent protein. In summary, this variant meets our criteria for pathogenicity.

DISEASE INFORMATION: Cystic fibrosis effects the epithelia of the respiratory tract, exocrine pancreas, intestine, male genital tract, hepatobiliary system, and exocrine sweat glands, resulting in a complex multisystem disease. Pulmonary disease is the major cause of morbidity and mortality in CF. Affected individuals have lower airway inflammation and chronic endobronchial infection, progressing to end-stage lung disease characterized by extensive airway damage (bronchiectasis, cysts, and abscesses) and fibrosis of lung parenchyma. Meconium ileus occurs at birth in 15%-20% of newborns with CF. Pancreatic insufficiency with malabsorption occurs in the great majority of individuals with CF. More than 95%of males with CF are infertile as a result of azoospermia caused by absent, atrophic, or fibrotic Wolffian duct structures. Adapted from GeneReviews abstract: http://www.ncbi.nlm.nih.gov/books/NBK1250/

FAMILIAL RISK: Cystic fibrosis (CF) due to pathogenic variants in the CFTR gene is inherited in an autosomal recessive manner. The risk of this patient's child having CF is dependent on the carrier status of the patient's partner. Two carriers have a 25% risk for having a child with CF. Other biologically related family members may also be carriers of this variant.

GENOME REPORT (CONTINUED)

Disease Inheritance	Gene Transcript	Zygosity Variant Classification	Variant Frequency	Disease Prevalence (Carrier Freq.)	References	Phenotype in carriers
Glycogen storage disease 7 Autosomal recessive	PFKM NM_000289.5	Heterozygous c.237+1G>A Pathogenic	Not previously reported	Unknown (Unknown)	Raben 1993	None reported

VARIANT INTERPRETATION: The 237+1G>A variant in PFKM has been previously identified in one homozygous patient with glycogen storage disease 7 and was found to segregate with disease in an affected homozygous relative (Raben 1993). This variant is located in the 5' splice region and computational tools do suggest an impact to splicing. In summary, this variant meets our criteria for pathogenicity.

DISEASE INFORMATION: Glycogen storage disease 7 is caused by a deficiency of muscle phosphofructokinase activity. Symptoms usually appear in adulthood and are characterized by exercise intolerance with muscle cramps that can be accompanied by attacks of myoglobinuria. Some patients also experience compensated hemolytic anemia and early onset myogenic hyperuricemia. In addition to the accumulation of normal glycogen in muscle, an abnormal glycogen, resembling amylopectin, can be found in some muscle fibers. Adapted from Online Metabolic and Molecular Basis of Inherited Disease abstract: http://www.ommbid.com//OMMBID/the_online_metabolic_and_molecular_bases_of_inherited_disease/b/abstract/part7/ch71

FAMILIAL RISK: Glycogen storage disease 7 (GSD7) due to pathogenic variants in PFKM is inherited in an autosomal recessive manner. The risk of this patient's child having GSD is dependent on the carrier status of the patient's partner. Two carriers have a 25% risk for having a child with GSD7. Other biologically related family members may also be carriers of this variant.

PHARMACOGENOMIC ASSOCIATIONS AND BLOOD GROUPS

C. PHARMACOGENOMIC ASSOCIATIONS

Drug (Indication)	Summary	Variants Evaluated and Genotypes Identified	Interpretation	References (PMID)
Warfarin (Anti-coagulation)	**Standard dose requirement**	*CYP2C9*<br>rs1799853<br>rs1057910<br>Genotype: *1/*2<br>c.[430C;1075A];<br>c.[430C>T;1075A]<br><br>*VKORC1*<br>rs9923231<br>Genotype: AA	Patients with the CYP2C9*1/*2 genotype may require a lower dose of warfarin as compared to patients with theCYP2C9*1/*1 genotype. Patients with theVKORC1 AA genotype may require a lower dose of warfarin as compared to patients with the VKORC1 GG or GA genotypes. However, patients with the combination of the CYP2C9*1/*2 genotype and VKORC1 AA genotype are predicted to require standard doses of warfarin compared to other patients. Refer to warfarindosing.org for dosing based on genotype and other clinical factors.	Johnson 2011

VKORC1/CYP2C9 genotype combination frequencies

Dosing Group	VKORC1 rs9923231	CYP2C9 Genotypes	Approximate Frequency
Lower	AA	*1/*3, *2/*2, *2/*3, *3/*3	6%
	GA	*2/*3, *3/*3	3%
Standard	AA	*1/*1, *1/*2	37%
	GA	*1/*2, *1/*3, *2/*2	14%
	GG	*1/*3, *2/*2, *2/*3	<1%
Higher	GA	*1/*1	28%
	GG	*1/*1, *1/*2	13%

Drug (Indication)	Summary	Variants Evaluated and Genotypes Identified	Interpretation	References (PMID)
Clopidogrel (Anti-coagulation)	**Typical response to clopidogrel**	*CYP2C19*<br>rs4244285<br>rs4986893<br>rs12248560<br><br>Genotype: *1/*1<br>c.[806C(;)681G(;)636G];[-806C(;)681G(;)636G]	Patients with the CYP2C19 *1/*1 genotype may have extensive (typical) metabolism of clopidogrel as well as well as typical response to clopidogrel as compared to ultrarapid or poor clopidogrel metabolizers. Additional information and dosing recommendations for this result can be found at: http://www.pharmgkb.org/drug/PA449053.	Scott 2013

CYP2C19 genotype frequencies

Metabolism	Genotypes	Frequency
Ultrarapid	*1/*17, *17/*17	5-30%
Extensive (typical)	*1/*1	35-50%
Intermediate	*1/*2, *1/*3, *2/17, *3/*17	18-35%
Poor	*2/*2, *2/*3, *3/*3	2-15%

Digoxin (Dysrhythmias, heart failure)	**Intermediate metabolism and serum concentration of digoxin**	*ABCB1*<br>rs1045642<br>Genotype: CT<br><br>*Genotype frequencies:*<br>*CC: 22%* ***CT: 51%*** *TT:27%*	Patients with the CT genotype who take oral digoxin may have intermediate metabolism and serum concentrations of digoxin as compared to patients with the CC and TT genotypes.	Aarnoudse 2008, Kurata 2002, Hoffmeyer 2000
Metformin (Type 2 diabetes mellitus)	**Typical glycemic response to metformin**	C11orf65<br>rs11212617<br>Genotype: TT<br><br>*Genotype frequencies:*<br>***TT:37%*** *TG:48% GG:15%*	Patients with the TT genotype who have Type 2 Diabetes Mellitus and are treated with metformin may have a decreased glycemic response as compared to patients with the GG genotype. An association with increased or decreased glycemic response to metformin was not seen in people diagnosed with impaired glucose tolerance in the absence of Type 2Diabetes Mellitus.	Florez 2012, GoDARTS and UKPDS Diabetes Pharmacogenetics Study Group 2011
Simvastatin (Hyperlipidemia)	**Typical risk of simvastatin-related myopathy**	SLCO1B1<br>rs4149056<br>Genotype: TT<br><br>*Genotype frequencies:*<br>***TT:68%*** *TC:30% CC:2%*	Patients with the TT genotype may have a lower risk of simvastatin-related myopathy as compared to patients with the CT or CC genotype.	Wilke 2012

D. RED BLOOD CELL AND PLATELET ANTIGENS

SUMMARY

ABO Rh Blood type: A Negative

Rare RBC Antigens

No rare presence or absence of RBC antigens was identified.

Rare Platelet Antigens

Antigen	Frequency	Comments
HPA-1(a-)	2%	Increased risk of alloantibody formation in individual. Very desirable antigen negative donor.

DISCUSSION

These red blood cell (RBC) and human platelet antigen (HPA) predictions are based on published genotype to phenotype correlations for the alleles present. Some antigens have also been serologically determined using traditional blood typing methods.

During pregnancy or transfusion alloantibodies to blood group antigens and platelet antigens can form against foreign RBCs that contain immunogenic blood group and platelet antigens that the recipient is missing. These alloantibodies can cause clinically important complications during future transfusions and pregnancy.

Blood Production Transfusion

This test revealed an absence of the high frequency platelet HPA-1a antigen, which is present in 98% of the population. Therefore this individual has an increased risk of forming an unusual and clinically significant anti-HPA-1a alloantibody that is associated with immune-mediated platelet transfusion refractoriness/clearance. In pregnant women, this may can cause destruction of mismatched fetal and neonatal platelets.

Blood Production Donation

This individual would be a rare and very desirable platelet donor given that only 2% of the population is HPA-1(a-). Anti-HPA-1a alloantibodies are the most common anti-HPA alloantibody cause of a life threatening destruction of fetal/neonatal platelets, known as Fetal/Neonatal alloimmune thrombocytopenia (FNAIT).

RED BLOOD CELL ANTIGENS

A	B	H	D	C	c	E	e	K	k	Jk(a)	Jk(b)	Fy(a)	Fy(b)
+	-	+	-	-	+	-	+	-	+	+	+	+	-

M	N	S	S	Lu(a)	Lu(b)	Au(a)	Au(b)	Kp(a)	Kp(b)	Kp(c)	Di(a)	Di(b)
+	-	+	-	[+]	[+]	[+]	[+]	[-]	[+]	[-]	[-]	[+]

Wr(a)	Wr(b)	Yt(a)	Yt(b)	Sc1	Sc2	Do(a)	Do(b)	Jo(a)	Hy	Co(a)	Co(b)	LW(a)	LW(b)
[-]	[+]	[+]	[-]	[+]	[-]	[+]	[-]	[+]	[+]	[+]	[-]	[+]	[-]

Cr(a)	Kn(a)	Kn(b)	Sl(a)	Vil	Yk(a)	KCAM	McC(a)	McC(b)	In(a)	In(b)
[+]	[+]	[-]	[+]	[-]	[+]	[+]	[+]	[-]	[-]	[+]

Ok(a)	MER2	JMHK	JMHL	FORS
[+]	[+]	[+]	[+]	[-]

PLATELET ANTIGENS

1a	1b	2a	2b	3a	3b	4a	4b	5a	5b	6bw	7bw	8bw	9bw
**[-]**	[+]	[+]	[-]	[+]	[-]	[+]	[-]	[+]	[-]	[-]	[-]	[-]	[-]

10bw	11bw	12bw	13bw	14bw	15a	15b	16bw	17bw	18bw	19bw	20bw	21bw	22bw
[-]	[-]	[-]	[-]	[-]	[+]	[+]	[-]	[-]	[-]	[-]	[-]	[-]	[-]

23bw	24bw	25bw	26bw	27bw
[-]	[-]	[-]	[-]	[-]

Key: [+] presence of antigen predicted by genotyping; + presence of antigen predicted by genotyping and confirmed by serology; +* presence of antigen detected by serology, genotype prediction not available; [+w] weak presence of antigen predicted by genotyping; +w weak presence of antigen predicted by genotyping and confirmed by serology; +w* weak presence of antigen detected by serology, genotype prediction not available; [−] absence of antigen predicted by genotyping; - absence of antigen predicted by genotyping and confirmed by serology, -* absence of antigen detected by serology, genotype prediction not available; NC indicates no sequencing coverage, Dis indicates discordant. Rare (less than 5% population frequency) presence or absence of antigen is indicated in **red**.

METHODOLOGY

Genomic sequencing is performed using next generation sequencing on the Illumina HiSeq platform. Genomes are sequenced to at least 30X mean coverage and a minimum of 95% of bases are sequenced to at least 8X coverage. Paired-end 100bp reads are aligned to the NCBI reference sequence (GRCh37) using the Burrows-Wheeler Aligner (BWA), and variant calls are made using the Genomic Analysis Tool Kit (GATK). Variants are subsequently filtered to identify: (1) variants classified as disease causing in public databases; (2) nonsense, frameshift, and +/-1,2 splice-site variants that are novel or have a minor allele frequency <1% in European American or African American chromosomes from the NHLBI Exome Sequencing Project (http://evs.gs.washington.edu/EVS/); and (3) rs11212617 (C11orf65; metformin), rs12248560 (CYP2C19; clopidogrel), rs4244285 (CYP2C19; clopidogrel), rs4986893 (CYP2C19; clopidogrel), rs28399504 (CYP2C19; clopidogrel), rs41291556 (CYP2C19; clopidogrel), rs72552267 (CYP2C19; clopidogrel), rs72558186 (CYP2C19; clopidogrel), rs56337013 (CYP2C19; clopidogrel), rs1057910 (CYP2C9; warfarin), rs1799853 (CYP2C9; warfarin), rs7900194 (CYP2C9; warfarin), rs9332131 (CYP2C9; warfarin), rs28371685 (CYP2C9; warfarin), rs28371686 (CYP2C9; warfarin), rs9923231 (VKORC1; warfarin), rs4149056 (SLCO1B1; simvastatin), and rs1045642 (ABCB1; digoxin). The evidence for phenotype-

causality is then evaluated for each variant resulting from the filtering strategies above and variants are classified according to LMM criteria (http://pcpgm.partners.org/LMM). Only those variants with evidence for causing highly penetrant disease or contributing to disease in a recessive manner are reported. Before reporting, all variants are confirmed via Sanger sequencing or another orthogonal technology. The initial sequencing component of this test was performed by the Illumina Clinical Services Laboratory (San Diego, CA CLIA# 05D1092911) and the alignment, variant calling, data filtering, Sanger confirmation and interpretation were performed by the Laboratory for Molecular Medicine at the Partners Healthcare Center for Personalized Genetic Medicine (Cambridge, MA CLIA#22D1005307). This test has not been cleared or approved U.S. Food and Drug Administration (FDA). The FDA has determined that such clearance or approval is not necessary.

LIMITATIONS

It should be noted that this test does not sequence all bases in a human genome and not all variants have been identified or interpreted. Triplet repeat expansions, translocations and large copy number events are currently not reliably detected by genome sequencing. Furthermore, not all disease-associated genes have been identified and the clinical significance of variation in many genes is not well understood. It is recommended that genomic sequencing data is periodically reinterpreted, especially when new symptoms arise.

COVERAGE OF ANALYZED GENES RELEVANT TO CARDIOVASCULAR DISEASE

The table below provides a list of genes relevant to cardiovascular disease that were evaluated during this individual's genome sequencing analysis. The proportion of the gene covered at ≥8X, *e.g.* the proportion of the gene with at least 8 mapped reads, is also provided. Please note that the presence of pathogenic variation in genes not analyzed or with incomplete coverage cannot be fully excluded.

Gene	Test Coverage >8X (%)[a]	HCM	DCM	ARVC	CPVT	LVNC	RCM	Relative contribution to HCM (%)[b]
**MYBPC3**	100	**X**	X			X		50%
**MYH7**	100	**X**	X			X	(X)	33%
**TNNI3**	100	**X**	X				(X)	5%
**TNNT2**	100	**X**	X			X	(X)	4%
**TPM1**	100	**X**	X					3%
**MYL2**	100	**X**						2%
**PRKAG2**	100	**X†**						1%
**GLA**	100	**X†**						1%
**MYL3**	100	**X**						1%
**LAMP2**	99.9	**X**	X					1%
**ACTC1**	100	**X**	X			X	(X)	0.4%
**PLN**	100	**X**	X					0.1%
**ACTN2**	100	**X**	X					unknown
**CSRP3**	100	**X**	X					unknown
**MYOZ2**	100	**X**						unknown
**NEXN**	100	**X**	X					unknown
**PTPN11**	99.9	**X†**						unknown
**RAF1**	100	**X†**						unknown
**TNNC1**	100	**X**	X					unknown
**TTR**	100	**X†**						unknown
ANKRD1	100	(X)	(X)					unknown
BAG3	100	(X)†	X				(X)†	unknown
CAV3	100	(X)	(X)					unknown
JPH2	99.8	(X)						unknown
LDB3	99.2	(X)	X			X		unknown
MYH6	100	(X)	(X)					unknown
MYLK2	100	(X)						unknown
MYOM1	100	(X)						unknown
MYPN	100	(X)						unknown
PDLIM3	100	(X)	(X)					unknown
RYR2	100	(X)		(X)	X			unknown
TCAP	100	(X)	X†					unknown
TTN	99.6	(X)	X	(X)				unknown
VCL	99.7	(X)	X			X		unknown
ABCC9	100		X					unknown
CASQ2	100				X	X		unknown
CHRM2	100		(X)					unknown
CRYAB	100		(X)					unknown
DES	100		X	(X)			(X)	unknown
DMD	99.2		X					unknown
DOLK	99.9		(X)					unknown
DSC2	100		(X)	X				unknown
DSG2	100		(X)	X				unknown
DSP	100		X	X				unknown
DTNA	100					X		unknown
EMD	96.8		X†					unknown
FHL2	100		(X)					unknown
GATAD1	99.9		X					unknown
ILK	100		(X)					unknown
JUP	100			X				unknown
LAMA4	100		(X)					unknown
LMNA	100		X			X		unknown
MURC	100		(X)					unknown
NEBL	100		(X)					unknown
PKP2	100		(X)	X				unknown
PRDM16	99.7		(X)					unknown
RBM20	100		X					unknown
SCN5A	100		X					unknown
SGCD	100		X†					unknown
TAZ	99.7		X†			X†		unknown
TMEM43	100			X				unknown
TRDN	100				X			unknown

X = genes with an established or likely role in the noted cardiomyopathy; X† = cardiomyopathy seen as part of larger disease spectrum; (X) = genes with limited evidence for disease association

[a]Indicates % coverage of gene at ≥8X in this patient's WGS analysis

[b]Based on LMM unpublished data

REFERENCES

Aarnoudse AJ, Dieleman JP, Visser LE, Arp PP, van der Heiden IP, van Schaik RH, Molokhia M, Hofman A, Uitterlinden AG, Stricker BH. 2008. Common ATP-binding cassette B1 variants are associated with increased digoxin serum concentration. Pharmacogenet. Genomics. 18(4):299-305.

Florez JC, Jablonski KA, Taylor A, Mather K, Horton E, White NH, Barrett-Connor E, Knowler WC, Shuldiner AR, Pollin TI, Diabetes Prevention Program Research Group. 2012. The C allele of ATM rs11212617 does not associate with metformin response in the Diabetes Prevention Program. Diabetes Care. 35(9):1864-7.

GoDARTS and UKPDS Diabetes Pharmacogenetics Study Group, Wellcome Trust Case Control Consortium 2, Zhou K, Bellenguez C, Spencer CC, Bennett AJ, Coleman RL, Tavendale R, Hawley SA, Donnelly LA, Schofield C, Groves CJ, Burch L, Carr F, Strange A, Freeman C, Blackwell JM, Bramon E, Brown MA, Casas JP, Corvin A, Craddock N, Deloukas P, Dronov S, Duncanson A, Edkins S, Gray E, Hunt S, Jankowski J, Langford C, Markus HS, Mathew CG, Plomin R, Rautanen A, Sawcer SJ, Samani NJ, Trembath R, Viswanathan AC, Wood NW, MAGIC investigators, Harries LW, Hattersley AT, Doney AS, Colhoun H, Morris AD, Sutherland C, Hardie DG, Peltonen L, McCarthy MI, Holman RR, Palmer CN, Donnelly P, Pearson ER. 2011. Common variants near ATM are associated with glycemic response to metformin in type 2 diabetes. Nat. Genet. 43(2):117-20.

Hamosh A, Trapnell BC, Zeitlin PL, Montrose-Rafizadeh C, Rosenstein BJ, Crystal RG, Cutting GR. 1991. Severe deficiency of cystic fibrosis transmembrane conductance regulator messenger RNA carrying nonsense mutations R553X and W1316X in respiratory epithelial cells of patients with cystic fibrosis. J. Clin. Invest. 88(6):1880-5.

Hoffmeyer S, Burk O, von Richter O, Arnold HP, Brockmöller J, Johne A, Cascorbi I, Gerloff T, Roots I, Eichelbaum M, Brinkmann U. 2000. Functional polymorphisms of the human multidrug-resistance gene: multiple sequence variations and correlation of one allele with P-glycoprotein expression and activity in vivo. Proc. Natl. Acad. Sci. U.S.A. 97(7):3473-8.

Johnson JA, Gong L, Whirl-Carrillo M, Gage BF, Scott SA, Stein CM, Anderson JL, Kimmel SE, Lee MT, Pirmohamed M, Wadelius M, Klein TE, Altman RB, Clinical Pharmacogenetics Implementation Consortium. 2011. Clinical Pharmacogenetics Implementation Consortium Guidelines for CYP2C9 and VKORC1 genotypes and warfarin dosing. Clin. Pharmacol. Ther. 90(4):625-9.

Kerem BS, Zielenski J, Markiewicz D, Bozon D, Gazit E, Yahav J, Kennedy D, Riordan JR, Collins FS, Rommens JM. 1990. Identification of mutations in regions corresponding to the two putative nucleotide (ATP)-binding folds of the cystic fibrosis gene. Proc. Natl. Acad. Sci. U.S.A. 87(21):8447-51.

Kurata Y, Ieiri I, Kimura M, Morita T, Irie S, Urae A, Ohdo S, Ohtani H, Sawada Y, Higuchi S, Otsubo K. 2002. Role of human MDR1 gene polymorphism in bioavailability and interaction of digoxin, a substrate of P-glycoprotein. Clin. Pharmacol. Ther. 72(2):209-19.

Lekanne Deprez RH, Muurling-Vlietman JJ, Hruda J, Baars MJ, Wijnaendts LC, Stolte-Dijkstra I, Alders M, van Hagen JM. 2006. Two cases of severe neonatal hypertrophic cardiomyopathy caused by compound heterozygous mutations in the MYBPC3 gene. J. Med. Genet. 43(10):829-32.

Raben N, Sherman J, Miller F, Mena H, Plotz P. 1993. A 5' splice junction mutation leading to exon deletion in an Ashkenazic Jewish family with phosphofructokinase deficiency (Tarui disease). J. Biol. Chem. 268(7):4963-7.

Scott SA, Sangkuhl K, Stein CM, Hulot JS, Mega JL, Roden DM, Klein TE, Sabatine MS, Johnson JA, Shuldiner AR, Clinical Pharmacogenetics Implementation Consortium. 2013. Clinical Pharmacogenetics Implementation Consortium guidelines for CYP2C19 genotype and clopidogrel therapy: 2013 update. Clin. Pharmacol. Ther. 94(3):317-23.

Shoshani T, Augarten A, Gazit E, Bashan N, Yahav Y, Rivlin Y, Tal A, Seret H, Yaar L, Kerem E. 1992. Association of a nonsense mutation (W1282X), the most common mutation in the Ashkenazi Jewish cystic fibrosis patients in Israel, with presentation of severe disease. Am. J. Hum. Genet. 50(1):222-8.

Tajsharghi H, Leren TP, Abdul-Hussein S, Tulinius M, Brunvand L, Dahl HM, Oldfors A. 2010. Unexpected myopathy associated with a mutation in MYBPC3 and misplacement of the cardiac myosin binding protein C. J. Med. Genet. 47(8):575-7.

Van Driest SL, Vasile VC, Ommen SR, Will ML, Tajik AJ, Gersh BJ, Ackerman MJ. 2004. Myosin binding protein C mutations and compound heterozygosity in hypertrophic cardiomyopathy. J. Am. Coll. Cardiol. 44(9):1903-10.

Vidaud M, Fanen P, Martin J, Ghanem N, Nicolas S, Goossens M. 1990. Three point mutations in the CFTR gene in French cystic fibrosis patients: identification by denaturing gradient gel electrophoresis. Hum. Genet. 85(4):446-9.

Wilke RA, Ramsey LB, Johnson SG, Maxwell WD, McLeod HL, Voora D, Krauss RM, Roden DM, Feng Q, Cooper-Dehoff RM, Gong L, Klein TE, Wadelius M, Niemi M, Clinical Pharmacogenomics Implementation Consortium (CPIC). 2012. The clinical pharmacogenomics implementation consortium: CPIC guideline for SLCO1B1 and simvastatin-induced myopathy. Clin. Pharmacol. Ther. 92(1):112-7.
