# Supplementary material for: A systematic approach to the reporting of medically relevant findings from whole genome sequencing
Source: BMC Med Genet. 2014 Dec 14;15:134. doi: 10.1186/s12881-014-0134-1 (PMC4342199; doi:10.1186/s12881-014-0134-1)
Supplement: Additional file 3: — Example Genome Report 2. Cardiomyopathy patient with variant of uncertain significance. [file 12881_2014_134_MOESM3_ESM.pdf]

LABORATORY FOR MOLECULAR MEDICINE
65 Landsdowne St, Cambridge, MA 02139
Phone: (617) 768-8500 / Fax: (617) 768-8513
http://pcpgm.partners.org/lmm

PARTNERS HEALTHCARE | PERSONALIZED MEDICINE | MASSACHUSETTS GENERAL HOSPITAL | BRIGHAM AND WOMEN'S HOSPITAL

Name: Doe, Jane | **Accession ID:** PMXX-12345
DOB: 12/34/5678 | **MRN:** 123456789 | **Family #:** F12345
Sex: Female | **Specimen:** Blood, Peripheral | **Referring physician:** MedSeq
Race: Caucasian | **Received:** 12/34/5678 | **Referring facility:** MedSeq
Indication for testing: Clinical diagnosis of dilated cardiomyopathy, MedSeq
Test: WGS-pnlA, SeqConV2, WGS-GGR

GENOME REPORT

RESULT SUMMARY

Sequencing of this individual's genome was performed and covered 95.3% of all positions at 8X coverage or higher, resulting in over 5.3 million variants compared to a reference genome. These data were analyzed to identify previously reported variants of potential clinical relevance as well as novel variants that could reasonably be assumed to cause disease (see methodology below). All results are summarized on page 1 with further details on subsequent pages.

I. RESULTS RELEVANT TO INDICATION FOR TESTING

For this patient with a diagnosis of cardiomyopathy, we reviewed all variants found in 62 genes with known association with hereditary cardiovascular disease and identified one variant of uncertain significance. More information is needed to determine if this variant contributes to disease.

Disease, Inheritance	Phenotype	Gene Transcript	Zygosity Variant	Classification
Dilated cardiomyopathy, Autosomal dominant	Ventricular chamber enlargement	RBM20 NM_001134363.1	Heterozygous c.2662G>A p.Asp888Asn	Uncertain significance

II. OTHER VARIANTS OF MEDICAL SIGNIFICANCE (INCIDENTAL FINDINGS)

A. MONOGENIC DISEASE RISK: 0 VARIANTS IDENTIFIED

This test did not identify any genetic variants that may be responsible for existing disease or the development of disease in this individual's lifetime.

B. CARRIER RISK: 2 VARIANTS IDENTIFIED

This test identified carrier status for 2 autosomal recessive disorders.

Disease, Inheritance	Phenotype	Gene Transcript	Zygosity Variant	Classification	Phenotype in carriers*
Alpha-1 Antitrypsin Deficiency Disorder, Autosomal recessive	Emphysema +/- liver disease	SERPINA1 NM_000295.4	Heterozygous c.1096G>A p.Glu366Lys	Pathogenic	None reported
Hepatic lipase deficiency, Autosomal recessive	Elevated plasma cholesterol and triglyceride levels	LIPC NM_000236.2	Heterozygous c.866C>T p.Ser289Phe	Uncertain significance: Favor pathogenic	Elevated HDL

As a carrier for recessive genetic variants, this individual is at higher risk for having a child with one or more of these highly penetrant disorders. To determine the risk for this individual's future children to be affected, the partner of this individual would also need to be tested for variants in these genes. Other biologically related family members may also be carriers of these variants. *Carriers for some recessive disorders may be at risk for certain phenotypes. Please see variant descriptions for more information.

C. PHARMACOGENOMIC ASSOCIATIONS

This test identified the following pharmacogenomic associations. Additional pharmacogenomic results may be requested, but will require additional molecular confirmation prior to disclosure.

Drug	Risk and Dosing Information
Warfarin	Standard dose requirement
Clopidogrel	Typical response to clopidogrel
Digoxin	Intermediate metabolism and serum concentration of digoxin
Metformin	Typical glycemic response to metformin
Simvastatin	Typical risk of simvastatin-related myopathy

D. RED BLOOD CELL AND PLATELET ANTIGENS

This test identified the ABO Rh blood type as B Negative. Additional blood group information is available at the end of the report.

GENOME REPORT (CONTINUED)

It should be noted that the disease risk section of this report is limited only to variants with strong evidence for causing highly penetrant disease, or contributing to highly penetrant disease in a recessive manner. Not all variants identified have been analyzed, and not all regions of the genome have been adequately sequenced. These results should be interpreted in the context of the patient's medical evaluation, family history, and racial/ethnic background. Please note that variant classification and/or interpretation may change over time if more information becomes available. For questions about this report, please contact the Genome Resource Center at GRC@partners.org

DETAILED VARIANT INFORMATION

I. RESULTS RELEVANT TO CLINICAL INDICATION

Disease, Inheritance	Gene Transcript	Zygosity Variant Classification	Variant Frequency	Disease Prevalence	References
Dilated cardiomyopathy, Autosomal dominant	RBM20 NM_001134363.1	Heterozygous c.2662G>A p.Asp888Asn Uncertain significance	Not in large population studies	~1/2,500	Refaat 2012

VARIANT INTERPRETATION: The Asp888Asn variant in RBM20 has been reported in 1 individual with DCM and was absent from 1200 control chromosomes (1000 Caucasian and 200 Black; Refaat 2012). Our laboratory has detected this variant in >5 individuals with clinical features of or a clinical diagnosis of DCM (LMM unpublished data). This variant has also been identified in 0.5% (3/570) of European chromosomes by the ClinSeq Project (dbSNP rs201370621). It was initially reported as being present in European American chromosomes from the NHLBI Exome Sequencing Project, but was then removed due to insufficient data quality (http://evs.gs.washington.edu/EVS/). Computational prediction tools and conservation analysis do not provide strong support for or against an impact to the protein. In summary, the clinical significance of the Asp888Asn variant is uncertain.

DISEASE INFORMATION: Dilated cardiomyopathy (DCM) is characterized by left ventricular enlargement and systolic dysfunction. DCM usually presents with any one of the following: Heart failure with symptoms of congestion and/or reduced cardiac output, arrhythmias and/or conduction system disease and thromboembolic disease including stroke. The incidence of DCM is currently underestimated. Familial dilated cardiomyopathy is principally caused by genetic mutations in genes that encode for cytoskeletal and sarcomeric proteins in the cardiac myocyte. Adapted from GeneReviews abstract: http://www.ncbi.nlm.nih.gov/books/NBK1309/.

FAMILIAL RISK: Dilated Cardiomyopathy due to pathogenic variants in the RBM20 gene is typically inherited in an autosomal dominant pattern. Each first-degree relative has a 50% chance of inheriting the variant and its risk for disease.

II. OTHER VARIANTS OF MEDICAL SIGNIFICANCE (INCIDENTAL FINDINGS)

A. MONOGENIC DISEASE RISK

This test did not identify any genetic variants that may be responsible for existing disease or the development of disease in this individual's lifetime.

B. CARRIER RISK

Disease, Inheritance	Gene Transcript	Zygosity Variant Classification	Variant Frequency	Disease Prevalence (Carrier Freq.)	References	Phenotype in Carriers
Alpha-1 Antitrypsin Deficiency Disorder, Autosomal recessive	SERPINA1 NM_000295.4	Heterozygous c.1096G>A p.Glu366Lys Pathogenic	1.6% (140/8,600) European American	1/5,000-1/7,000 European American (1/60)	Stoller 2012	None Reported

VARIANT INTERPRETATION: The p.Glu366Lys variant in SERPINA1 (also known as p.Glu342Lys or PI*Z) is the most common alpha-1 antitrypsin deficiency allele, leading to a high risk of emphysema (and to a lesser extent liver disease) when homozygous. In summary, even with the high population frequency of this variant, it meets our criteria to be classified as pathogenic.

DISEASE INFORMATION: Alpha-1 Antitrypsin Deficiency Disorder (AATD) is one of the most common metabolic disorders in persons of northern European heritage, occurring in approximately one in 5,000-7,000 individuals in North America and one in 1,500-3,000 in Scandinavians. COPD, specifically emphysema, is the most common manifestation of AATD and smoking is the major factor influencing age of onset and course of disease. Some individuals also present with liver disease. AATD is caused by homozygosity for the common deficiency allele, PI*Z, of SERPINA1. Clinical manifestations are infrequent in heterozygotes, except in some smokers. Adapted from GeneReviews: http://www.ncbi.nlm.nih.gov/books/NBK1519/

FAMILIAL RISK: AATD is inherited in an autosomal recessive manner. The risk of this patient's child having AATD is dependent on the carrier status of the patient's partner. Two carriers have a 25% risk for having a child with AATD. Other biologically related family members may also be carriers of this variant.

GENOME REPORT (CONTINUED)

Disease, Inheritance	Gene Transcript	Zygosity Variant Classification	Variant Frequency	Disease Prevalence (Carrier Freq.)	References	Phenotype in Carriers
Hepatic lipase deficiency, Autosomal recessive	LIPC NM_000236.2	Heterozygous c.866C>T p.Ser289Phe Uncertain significance: Favor pathogenic	0.13% (11/8,584) European American	Unknown (Unknown)	Hegele 1991 Durstenfeld 1994	Elevated HDL

VARIANT INTERPRETATION: The p.Ser289Phe variant in LIPC has been reported in 1 compound heterozygous individual with hepatic lipase deficiency and segregated with disease in 3 affected compound heterozygous relatives from 1 family (Hegele 1991). This variant has been identified in 0.13% (11/8584) of European American chromosomes and 0.05% (4/4384) of African American chromosomes by the NHLBI Exome Sequencing Project (http://evs.gs.washington.edu/EVS/; dbSNP rs121912502). Although this variant has been seen in the general population, its frequency is low enough to be consistent with a recessive carrier frequency. In vitro assays indicate the p.Ser289Phe variant leads to reduced LIPC activity (Durstenfeld 1994). However, these types of assays may not accurately represent biological function. Computational prediction tools and conservation analysis also suggest that the p.Ser289Phe variant may impact the protein, though this information is not predictive enough to determine pathogenicity. In summary, while there is some suspicion for a pathogenic role, the clinical significance of the p.Ser289Phe variant is uncertain.

DISEASE INFORMATION: Hepatic lipase deficiency (HLD) is characterized by elevated plasma cholesterol and triglyceride levels. Premature atherosclerosis has been reported in some individuals with HLD. Carriers for HLD may have elevated HDL cholesterol levels.

FAMILIAL RISK: HLD due to mutations in the LIPC gene is typically inherited in an autosomal recessive manner. The risk of this patient's child having hepatic lipase deficiency is dependent on the carrier status of the patient's partner. Two carriers have a 25% risk for having a child with the disease. Other biologically related family members may also be carriers of this variant.

PHARMACOGENOMIC ASSOCIATIONS AND BLOOD GROUPS

C. PHARMACOGENOMIC ASSOCIATIONS

Drug (Indication)	Summary	Variants Evaluated and Genotypes Identified	Interpretation	References (PMID)
Warfarin (Anti-coagulation)	**Standard dose requirement**	*CYP2C9*<br>rs1799853<br>rs1057910<br>Genotype: *1/*2<br>c.[430C;1075A];<br>c.[430C>T;1075A]<br><br>*VKORC1*<br>rs9923231<br>Genotype: AA	Patients with the CYP2C9*1/*2 genotype may require a lower dose of warfarin as compared to patients with theCYP2C9*1/*1 genotype. Patients with theVKORC1 AA genotype may require a lower dose of warfarin as compared to patients with the VKORC1 GG or GA genotypes. However, patients with the combination of the CYP2C9*1/*2 genotype and VKORC1 AA genotype are predicted to require standard doses of warfarin compared to other patients. Refer to warfarindosing.org for dosing based on genotype and other clinical factors.	Johnson 2011

VKORC1/CYP2C9 genotype combination frequencies

Dosing Group	VKORC1 rs9923231	CYP2C9 Genotypes	Approximate Frequency
Lower	AA	*1/*3, *2/*2, *2/*3, *3/*3	6%
	GA	*2/*3, *3/*3	3%
Standard	AA	*1/*1, *1/*2	37%
	GA	*1/*2, *1/*3, *2/*2	14%
	GG	*1/*3, *2/*2, *2/*3	<1%
Higher	GA	*1/*1	28%
	GG	*1/*1, *1/*2	13%

Drug (Indication)	Summary	Variants Evaluated and Genotypes Identified	Interpretation	References (PMID)
Clopidogrel (Anti-coagulation)	**Typical response to clopidogrel**	*CYP2C19*<br>rs4244285<br>rs4986893<br>rs12248560<br><br>Genotype: *1/*1<br>c.[806C(;)681G(;)636G];[-806C(;)681G(;)636G]	Patients with the CYP2C19 *1/*1 genotype may have extensive (typical) metabolism of clopidogrel as well as well as typical response to clopidogrel as compared to ultrarapid or poor clopidogrel metabolizers. Additional information and dosing recommendations for this result can be found at: http://www.pharmgkb.org/drug/PA449053.	Scott 2013

CYP2C19 genotype frequencies

Metabolism	Genotypes	Frequency
Ultrarapid	*1/*17, *17/*17	5-30%
Extensive (typical)	*1/*1	35-50%
Intermediate	*1/*2, *1/*3, *2/17, *3/*17	18-35%
Poor	*2/*2, *2/*3, *3/*3	2-15%

Digoxin (Dysrhythmias, heart failure)	**Intermediate metabolism and serum concentration of digoxin**	*ABCB1*<br>rs1045642<br>Genotype: CT<br><br>*Genotype frequencies:*<br>*CC: 22%* ***CT: 51%*** *TT:27%*	Patients with the CT genotype who take oral digoxin may have intermediate metabolism and serum concentrations of digoxin as compared to patients with the CC and TT genotypes.	Aarnoudse 2008, Kurata 2002, Hoffmeyer 2000
Metformin (Type 2 diabetes mellitus)	**Typical glycemic response to metformin**	C11orf65<br>rs11212617<br>Genotype: TT<br><br>*Genotype frequencies:*<br>***TT:37%*** *TG:48% GG:15%*	Patients with the TT genotype who have Type 2 Diabetes Mellitus and are treated with metformin may have a decreased glycemic response as compared to patients with the GG genotype. An association with increased or decreased glycemic response to metformin was not seen in people diagnosed with impaired glucose tolerance in the absence of Type 2Diabetes Mellitus.	Florez 2012, GoDARTS and UKPDS Diabetes Pharmacogenetics Study Group 2011
Simvastatin (Hyperlipidemia)	**Typical risk of simvastatin-related myopathy**	SLCO1B1<br>rs4149056<br>Genotype: TT<br><br>*Genotype frequencies:*<br>***TT:68%*** *TC:30% CC:2%*	Patients with the TT genotype may have a lower risk of simvastatin-related myopathy as compared to patients with the CT or CC genotype.	Wilke 2012

D. RED BLOOD CELL AND PLATELET ANTIGENS

SUMMARY

ABO Rh Blood type: B Negative

Rare RBC Antigens

No rare presence or absence of RBC antigens was identified.

Rare Platelet Antigens

No rare presence or absence of platelet antigens was identified.

DISCUSSION

These red blood cell (RBC) and human platelet antigen (HPA) predictions are based on published genotype to phenotype correlations for the alleles present. Some antigens have also been serologically determined using traditional blood typing methods.
During pregnancy or transfusion alloantibodies to blood group antigens and platelet antigens can form against foreign RBCs that contain immunogenic blood group and platelet antigens that the recipient is missing. These alloantibodies can cause clinically important complications during future transfusions and pregnancy.

Blood Production Transfusion

This individual does NOT have an increased risk of forming unusual RBC or platelet alloantibodies, since this test revealed a normal presence of high frequency antigens and no antigen gene rearrangements.

GENOME REPORT (CONTINUED)

Blood Production Donation

This individual does NOT pose an increased risk to blood product recipients since this test revealed a normal presence of high frequency antigens and no antigen gene rearrangements.

RED BLOOD CELL ANTIGENS

A	B	H	D	C	c	E	e	K	k	Jk(a)	Jk(b)	Fy(a)	Fy(b)
-	+	+	-	-	+	-	+	-	+	+	+	+	-

M	N	S	s	Lu(a)	Lu(b)	Au(a)	Au(b)	Kp(a)	Kp(b)	Kp(c)	Di(a)	Di(b)
+	-	+	-	[+]	[+]	[+]	[+]	[-]	[+]	[-]	[-]	[+]

Wr(a)	Wr(b)	Yt(a)	Yt(b)	Sc1	Sc2	Do(a)	Do(b)	Jo(a)	Hy	Co(a)	Co(b)	LW(a)	LW(b)
[-]	[+]	[+]	[-]	[+]	[-]	[+]	[-]	[+]	[+]	[+]	[-]	[+]	[-]

Cr(a)	Kn(a)	Kn(b)	Sl(a)	Vil	Yk(a)	KCAM	McC(a)	McC(b)	In(a)	In(b)
[+]	[+]	[-]	[+]	[-]	[+]	[+]	[+]	[-]	[-]	[+]

Ok(a)	MER2	JMHK	JMHL	FORS
[+]	[+]	[+]	[+]	[-]

PLATELET ANTIGENS

1a	1b	2a	2b	3a	3b	4a	4b	5a	5b	6bw	7bw	8bw	9bw
[+]	[+]	[+]	[-]	[+]	[-]	[+]	[-]	[+]	[-]	[-]	[-]	[-]	[-]

10bw	11bw	12bw	13bw	14bw	15a	15b	16bw	17bw	18bw	19bw	20bw	21bw	22bw
[-]	[-]	[-]	[-]	[-]	[+]	[+]	[-]	[-]	[-]	[-]	[-]	[-]	[-]

23bw	24bw	25bw	26bw	27bw
[-]	[-]	[-]	[-]	[-]

Key: [+] presence of antigen predicted by genotyping; + presence of antigen predicted by genotyping and confirmed by serology; +* presence of antigen detected by serology, genotype prediction not available; [+w] weak presence of antigen predicted by genotyping; +w weak presence of antigen predicted by genotyping and confirmed by serology; +w* weak presence of antigen detected by serology, genotype prediction not available; [−] absence of antigen predicted by genotyping; - absence of antigen predicted by genotyping and confirmed by serology, -* absence of antigen detected by serology, genotype prediction not available; NC indicates no sequencing coverage, Dis indicates discordant. Rare (less than 5% population frequency) presence or absence of antigen is indicated in red.

METHODOLOGY

Genomic sequencing is performed using next generation sequencing on the Illumina HiSeq platform. Genomes are sequenced to at least 30X mean coverage and a minimum of 95% of bases are sequenced to at least 8X coverage. Paired-end 100bp reads are aligned to the NCBI reference sequence (GRCh37) using the Burrows-Wheeler Aligner (BWA), and variant calls are made using the Genomic Analysis Tool Kit (GATK). Variants are subsequently filtered to identify: (1) variants classified as disease causing in public databases; (2) nonsense, frameshift, and +/-1,2 splice-site variants that are novel or have a minor allele frequency <1% in European American or African American chromosomes from the NHLBI Exome Sequencing Project (http://evs.gs.washington.edu/EVS/); and (3) rs11212617 (C11orf65; metformin), rs12248560 (CYP2C19; clopidogrel), rs4244285 (CYP2C19; clopidogrel), rs4986893 (CYP2C19; clopidogrel), rs28399504 (CYP2C19; clopidogrel), rs41291556 (CYP2C19; clopidogrel), rs72552267 (CYP2C19; clopidogrel), rs72558186 (CYP2C19; clopidogrel), rs56337013 (CYP2C19; clopidogrel), rs1057910 (CYP2C9; warfarin), rs1799853 (CYP2C9; warfarin), rs7900194 (CYP2C9; warfarin), rs9332131 (CYP2C9; warfarin), rs28371685 (CYP2C9; warfarin), rs28371686 (CYP2C9; warfarin), rs9923231 (VKORC1; warfarin), rs4149056 (SLCO1B1; simvastatin), and rs1045642 (ABCB1; digoxin). The evidence for phenotype-causality is then evaluated for each variant resulting from the filtering strategies above and variants are classified according to LMM criteria (http://pcpgm.partners.org/LMM). Only those variants with evidence for causing highly penetrant disease or contributing to disease in a recessive manner are reported. Before reporting, all variants are confirmed via Sanger sequencing or another orthogonal technology. The initial sequencing component of this test was performed by the Illumina Clinical Services Laboratory (San Diego, CA CLIA# 05D1092911) and the alignment, variant calling, data filtering, Sanger confirmation and interpretation were performed by the Laboratory for Molecular Medicine at the Partners Healthcare Center for Personalized Genetic Medicine (Cambridge, MA

CLIA#22D1005307). This test has not been cleared or approved U.S. Food and Drug Administration (FDA). The FDA has determined that such clearance or approval is not necessary.

LIMITATIONS

It should be noted that this test does not sequence all bases in a human genome and not all variants have been identified or interpreted. Triplet repeat expansions, translocations and large copy number events are currently not reliably detected by genome sequencing. Furthermore, not all disease-associated genes have been identified and the clinical significance of variation in many genes is not well understood. It is recommended that genomic sequencing data is periodically reinterpreted, especially when new symptoms arise.

COVERAGE OF ANALYZED GENES RELEVANT TO CARDIOVASCULAR DISEASE

The table below provides a list of genes relevant to cardiovascular disease that were evaluated during this individual’s genome sequencing analysis. The proportion of the gene covered at ≥8X, *e.g.* the proportion of the gene with at least 8 mapped reads, is also provided. Please note that the presence of pathogenic variation in genes not analyzed or with incomplete coverage cannot be fully excluded.

Gene	Test Coverage >8X (%)[a]	DCM	HCM	ARVC	CPVT	LVNC	RCM	Relative contribution to DCM (%)[b]
**TTN**	100	**X**	(X)	(X)				12%
**LMNA**	99.9	**X**				X		4%
**MYH7**	100	**X**	X			X	(X)	3%
**TNNT2**	100	**X**	X			X	(X)	3%
**DSP**	100	**X**		X				2%
**TPM1**	100	**X**	X					2%
**RBM20**	99.4	**X**						1%
**VCL**	100	**X**	(X)			X		0.70%
**DES**	99.6	**X**		(X)			(X)	0.50%
**TAZ**	100	**X†**				X†		0.30%
**TNNI3**	100	**X**	X				(X)	0.30%
**ABCC9**	100	**X**						0.20%
**CSRP3**	100	**X**	X					0.20%
**PLN**	100	**X**	X					0.10%
**ACTC1**	100	**X**	X			X	(X)	unknown
**ACTN2**	100	**X**	X					unknown
**BAG3**	100	**X**	(X)†				(X)†	unknown
**MYBPC3**	100	**X**	X			X		unknown
**NEXN**	100	**X**	X					unknown
**DMD**	100	**X**						unknown
**EMD**	99.9	**X†**						unknown
**GATAD1**	99.4	**X**						unknown
**LAMP2**	100	**X**	X					unknown
**LDB3**	97.5	**X**	(X)			X		unknown
**SCN5A**	100	**X**						unknown
**SGCD**	100	**X†**						unknown
**TCAP**	100	**X†**	(X)					unknown
**TNNC1**	100	**X**	X					unknown
ANKRD1	99.9	(X)	(X)					unknown
CAV3	100	(X)	(X)					unknown
CHRM2	100	(X)						unknown

Gene	Test Coverage >8X (%)[a]	DCM	HCM	ARVC	CPVT	LVNC	RCM	Relative contribution to DCM (%)[b]
CRYAB	100	(X)						unknown
DOLK	100	(X)						unknown
DSC2	100	(X)		X				unknown
DSG2	100	(X)		X				unknown
FHL2	100	(X)						unknown
ILK	100	(X)						unknown
LAMA4	100	(X)						unknown
MURC	100	(X)						unknown
MYH6	100	(X)	(X)					unknown
NEBL	100	(X)						unknown
PDLIM3	100	(X)	(X)					unknown
PKP2	100	(X)		X				unknown
PRDM16	100	(X)						unknown
CASQ2	100				X	X		unknown
DTNA	100					X		unknown
GLA	100		X†					unknown
JPH2	99.6		(X)					unknown
JUP	100			X				unknown
MYL2	100		X					unknown
MYL3	100		X					unknown
MYLK2	100		(X)					unknown
MYOM1	100		(X)					unknown
MYOZ2	100		X					unknown
MYPN	100		(X)					unknown
PRKAG2	100		X†					unknown
PTPN11	100		X†					unknown
RAF1	100		X†					unknown
RYR2	100		(X)	(X)	X			unknown
TMEM43	100			X				unknown
TRDN	100				X			unknown
TTR	100		X†					unknown

X = genes with an established or likely role in the noted cardiomyopathy; X† = cardiomyopathy seen as part of larger disease spectrum; (X) = genes with limited evidence for disease association

[a]Indicates % coverage of gene at ≥8X in this patient's WGS analysis

[b]Based on LMM unpublished data

REFERENCES

Aarnoudse AJ, Dieleman JP, Visser LE, Arp PP, van der Heiden IP, van Schaik RH, Molokhia M, Hofman A, Uitterlinden AG, Stricker BH. 2008. Common ATP-binding cassette B1 variants are associated with increased digoxin serum concentration. Pharmacogenet. Genomics. 18(4):299-305.

Durstenfeld A, Ben-Zeev O, Reue K, Stahnke G, Doolittle MH. 1994. Molecular characterization of human hepatic lipase deficiency. In vitro expression of two naturally occurring mutations. Arterioscler. Thromb. 14(3):381-5.

Florez JC, Jablonski KA, Taylor A, Mather K, Horton E, White NH, Barrett-Connor E, Knowler WC, Shuldiner AR, Pollin TI, Diabetes Prevention Program Research Group. 2012. The C allele of ATM rs11212617 does not associate with metformin response in the Diabetes Prevention Program. Diabetes Care. 35(9):1864-7.

GoDARTS and UKPDS Diabetes Pharmacogenetics Study Group, Wellcome Trust Case Control Consortium 2, Zhou K, Bellenguez C, Spencer CC, Bennett AJ, Coleman RL, Tavendale R, Hawley SA, Donnelly LA, Schofield C, Groves CJ, Burch L, Carr F, Strange A, Freeman C, Blackwell JM, Bramon E, Brown MA, Casas JP, Corvin A, Craddock N, Deloukas P, Dronov S, Duncanson A, Edkins S, Gray E, Hunt S, Jankowski J, Langford C, Markus HS, Mathew CG, Plomin R, Rautanen A, Sawcer SJ, Samani NJ, Trembath R, Viswanathan AC, Wood NW, MAGIC investigators, Harries LW, Hattersley AT, Doney AS, Colhoun H, Morris AD, Sutherland C, Hardie DG, Peltonen L, McCarthy MI, Holman RR, Palmer CN, Donnelly P, Pearson ER. 2011. Common variants near ATM are associated with glycemic response to metformin in type 2 diabetes. Nat. Genet. 43(2):117-20.

Hegele RA, Little JA, Connelly PW. 1991. Compound heterozygosity for mutant hepatic lipase in familial hepatic lipase deficiency. Biochem. Biophys. Res. Commun. 179(1):78-84.

Hoffmeyer S, Burk O, von Richter O, Arnold HP, Brockmöller J, Johne A, Cascorbi I, Gerloff T, Roots I, Eichelbaum M, Brinkmann U. 2000. Functional polymorphisms of the human multidrug-resistance gene: multiple sequence variations and correlation of one allele with P-glycoprotein expression and activity in vivo. Proc. Natl. Acad. Sci. U.S.A. 97(7):3473-8.

Johnson JA, Gong L, Whirl-Carrillo M, Gage BF, Scott SA, Stein CM, Anderson JL, Kimmel SE, Lee MT, Pirmohamed M, Wadelius M, Klein TE, Altman RB, Clinical Pharmacogenetics Implementation Consortium. 2011. Clinical Pharmacogenetics Implementation Consortium Guidelines for CYP2C9 and VKORC1 genotypes and warfarin dosing. Clin. Pharmacol. Ther. 90(4):625-9.

Kurata Y, Ieiri I, Kimura M, Morita T, Irie S, Urae A, Ohdo S, Ohtani H, Sawada Y, Higuchi S, Otsubo K. 2002. Role of human MDR1 gene polymorphism in bioavailability and interaction of digoxin, a substrate of P-glycoprotein. Clin. Pharmacol. Ther. 72(2):209-19.

Refaat MM, Lubitz SA, Makino S, Islam Z, Frangiskakis JM, Mehdi H, Gutmann R, Zhang ML, Bloom HL, MacRae CA, Dudley SC, Shalaby AA, Weiss R, McNamara DM, London B, Ellinor PT. 2012. Genetic variation in the alternative splicing regulator RBM20 is associated with dilated cardiomyopathy. Heart Rhythm. 9(3):390-6.

Scott SA, Sangkuhl K, Stein CM, Hulot JS, Mega JL, Roden DM, Klein TE, Sabatine MS, Johnson JA, Shuldiner AR, Clinical Pharmacogenetics Implementation Consortium. 2013. Clinical Pharmacogenetics Implementation Consortium guidelines for CYP2C19 genotype and clopidogrel therapy: 2013 update. Clin. Pharmacol. Ther. 94(3):317-23.

Stoller JK, Aboussouan LS. 2012. A review of α1-antitrypsin deficiency. Am. J. Respir. Crit. Care Med. 185(3):246-59.

Wilke RA, Ramsey LB, Johnson SG, Maxwell WD, McLeod HL, Voora D, Krauss RM, Roden DM, Feng Q, Cooper-Dehoff RM, Gong L, Klein TE, Wadelius M, Niemi M, Clinical Pharmacogenomics Implementation Consortium (CPIC). 2012. The clinical pharmacogenomics implementation consortium: CPIC guideline for SLCO1B1 and simvastatin-induced myopathy. Clin. Pharmacol. Ther. 92(1):112-7.
