# Supplementary material for: A systematic approach to the reporting of medically relevant findings from whole genome sequencing
Source: BMC Med Genet. 2014 Dec 14;15:134. doi: 10.1186/s12881-014-0134-1 (PMC4342199; doi:10.1186/s12881-014-0134-1)
Supplement: Additional file 4: — Example Genome Report 3. Primary care patient with risk allele. [file 12881_2014_134_MOESM4_ESM.pdf]

LABORATORY FOR MOLECULAR MEDICINE
65 Landsdowne St, Cambridge, MA 02139
Phone: (617) 768-8500 / Fax: (617) 768-8513
http://pcpgm.partners.org/lmm

PARTNERS HEALTHCARE | PERSONALIZED MEDICINE | MASSACHUSETTS GENERAL HOSPITAL | BRIGHAM AND WOMEN'S HOSPITAL

**Name: Doe, Jeffrey**
DOB: **12/34/5678**
Sex: **Male**
Race: **Caucasian**
Indication for testing: **MedSeq**
Test: **WGS-pnlA, SeqConV2, WGS-GGR**

MRN: **123456789**
Specimen: **Blood, Peripheral**
Received: **12/34/5678**

**Accession ID: PMXX-12345**
Family #: **F12345**
Referring physician: **MedSeq**
Referring facility: **MedSeq**

# GENOME REPORT

## RESULT SUMMARY

Sequencing of this individual's genome was performed and covered 95.7% of all positions at 8X coverage or higher, resulting in over 5.4 million variants compared to a reference genome. These data were analyzed to identify previously reported variants of potential clinical relevance as well as novel variants that could reasonably be assumed to cause disease (see methodology below). All results are summarized on page 1 with further details on subsequent pages.

### A. MONOGENIC DISEASE RISK: 1 VARIANT IDENTIFIED

| Disease, Inheritance | Phenotype | Gene Transcript | Zygosity Variant | Classification |
|---|---|---|---|---|
| Factor V Leiden thrombophilia, Multi-factorial | Venous thromboembolism | F5 NM_000130.4 | Heterozygous c.1601G>A p.Arg534Gln | Risk allele |

### B. CARRIER RISK: 2 VARIANTS IDENTIFIED

This test identified carrier status for 2 autosomal recessive disorders.

| Disease, Inheritance | Phenotype | Gene Transcript | Zygosity Variant | Classification | Phenotype in carriers * |
|---|---|---|---|---|---|
| Achromatopsia, Autosomal recessive | Color blindness | CNGA3 NM_001298.2 | Heterozygous c.101+1G>A | Likely pathogenic | None reported |
| Hereditary hemochromatosis, Autosomal recessive | Excessive iron storage | HFE NM_000410.3 | Heterozygous c.187C>G p.His63Asp | Pathogenic | None reported |

As a carrier for recessive genetic variants, this individual is at higher risk for having a child with one or more of these highly penetrant disorders. To determine the risk for this individual's future children to be affected, the partner of this individual would also need to be tested for variants in these genes. Other biologically related family members may also be carriers of these variants. *Carriers for some recessive disorders may be at risk for certain phenotypes. Please see variant descriptions for more information.

### C. PHARMACOGENOMIC ASSOCIATIONS

This test identified the following pharmacogenomic associations. Additional pharmacogenomic results may be requested, but will require additional molecular confirmation prior to disclosure.

| Drug | Risk and Dosing Information |
|---|---|
| Warfarin | Standard dose requirement |
| Clopidogrel | Typical response to clopidogrel |
| Digoxin | Intermediate metabolism and serum concentration of digoxin |
| Metformin | Typical glycemic response to metformin |
| Simvastatin | Typical risk of simvastatin-related myopathy |

### D. RED BLOOD CELL AND PLATELET ANTIGENS

This test identified the ABO Rh blood type as B Negative. Additional blood group information is available at the end of the report.

It should be noted that the disease risk section of this report is limited only to variants with strong evidence for causing highly penetrant disease, or contributing to highly penetrant disease in a recessive manner. Not all variants identified have been analyzed, and not all regions of the genome have been adequately sequenced. These results should be interpreted in the context of the patient's medical evaluation, family history, and racial/ethnic background. Please note that variant classification and/or interpretation may change over time if more information becomes available. For questions about this report, please contact the Genome Resource Center at GRC@partners.org

**GENOME REPORT (CONTINUED)**

# DETAILED VARIANT INFORMATION

## A. MONOGENIC DISEASE RISK

| Disease, Inheritance | Gene Transcript | Zygosity Variant Classification | Variant Frequency | Disease Prevalence | References |
|---|---|---|---|---|---|
| Factor V Leiden Thrombophilia, Multi-factorial | F5 NM_000130.4 | Heterozygous c.1601G>A p.Arg534Gln Risk allele | 3-8% European American | Unknown | Kujovich 2011 Simone 2013 |

**VARIANT INTERPRETATION:** The Arg534Gln (legacy name Arg506Gln) in F5 is commonly referred to as "factor V Leiden" and has been associated with increased risk for venous thromboembolism (VTE) with an overall odds ratio (OR) of 4.3 (Simone 2013). The frequency of this variant varies by population, with the highest heterozygosity rate found in Europe. It has been identified in 3% (259/8600) of European American chromosomes and 0.4% (19/4406) of African American chromosomes by the NHLBI Exome Sequencing Project (http://evs.gs.washington.edu/EVS/; dbSNP rs6025). The factor V Leiden mutation is present in approximately 15-20% of individuals with a first deep vein thrombosis (DVT) and in up to 50% of individual with recurrent VTE or an estrogen-related thrombosis. Many individuals with the factor V Leiden allele never develop thrombosis, however, evidence suggests that the relative risk for VTE is increased 3- to 8-fold in factor V Leiden heterozygotes and 10- to 80-fold in homozygotes. Lastly, heterozygosity for factor V Leiden is associated with a 2- to 3-fold increase in relative risk for pregnancy loss, and possibly other pregnancy complications such as preeclampsia, fetal growth retardation, and placental abruption.

**DISEASE INFORMATION:** Factor V Leiden thrombophilia is the most common inherited form of thrombophilia. It is characterized by a poor anticoagulant response to activated protein C (APC) and an increased risk for venous thromboembolism (VTE), which is a common complex (multifactorial) disease. Deep venous thrombosis (DVT) is the most common VTE, with the legs being the most common site. Thrombosis in unusual locations is less common. The clinical expression of factor V Leiden thrombophilia is influenced by the number of factor V Leiden alleles, coexisting genetic or acquired thrombophilic disorders and circumstantial risk factors (e.g., travel, pregnancy, oral contraceptives, central venous catheters, advancing age and surgery). Adapted from GeneReviews: http://www.ncbi.nlm.nih.gov/books/NBK1368/.

**FAMILIAL RISK:** This Factor V Leiden risk allele increases the chance of developing thrombophilia, likely in conjunction with other genetic and/or environmental risk factors. For a heterozygous individual, there is a 50% chance of passing on the variant and its risk of thrombophilia to a first degree relative. For homozygous individuals, there is a 100% chance of passing on the variant and its risk of thrombophilia to a first degree relative.

## B. CARRIER RISK

| Disease, Inheritance | Gene Transcript | Zygosity Variant Classification | Variant Frequency | Disease Prevalence (Carrier Freq.) | References | Phenotype in carriers |
|---|---|---|---|---|---|---|
| Achromatopsia, Autosomal recessive | CNGA3 NM_001298.2 | Heterozygous c.101+1G>A Likely pathogenic | 0.08% (7/8600) European American | <1/30,000 (Unknown) | Wissinger 2001 | None reported |

**VARIANT INTERPRETATION:** The c.101+1G>A variant in CNGA3 has not been previously reported in individuals with achromatopsia, but has been identified in 0.08% (7/8600) of European American chromosomes and 0.02% (1/4406) of African American chromosomes by the NHLBI Exome Sequencing Project (http://evs.gs.washington.edu/EVS/; dbSNP rs147118493). Although this variant has been seen in the general population, its frequency is low enough to be consistent with a recessive carrier frequency. This variant occurs in the invariant region (+/- 1,2) of the splice consensus sequence and is predicted to cause altered splicing leading to an abnormal or absent protein. Complete loss of CNGA3 function is an established disease mechanism in individuals with achromatopsia (Wissinger 2001). In summary, although additional studies are required to fully establish its clinical significance, the c.101+1G>A variant is likely pathogenic.

**DISEASE INFORMATION:** Achromatopsia is characterized by reduced visual acuity, pendular nystagmus, increased sensitivity to light (photophobia), a small central scotoma, eccentric fixation, and reduced or complete loss of color discrimination. Individuals with achromatopsia have impaired color discrimination along all three axes of color vision (red, green, and blue). Adapted from GeneReviews abstract: http://www.ncbi.nlm.nih.gov/books/NBK1418/

**FAMILIAL RISK:** Achromatopsia is inherited in an autosomal recessive manner. The risk of this patient’s child having the disease is dependent on the carrier status of the patient’s partner. Two carriers have a 25% risk for having a child with achromatopsia. Other biologically related family members may also be carriers of this variant.

**GENOME REPORT (CONTINUED)**

| Disease, Inheritance | Gene Transcript | Zygosity Variant Classification | Variant Frequency | Disease Prevalence (Carrier Freq.) | References | Phenotype in Carriers |
|---|---|---|---|---|---|---|
| Hereditary hemochromatosis, Autosomal recessive | HFE NM_000410.3 | Heterozygous c.187C>G p.His63Asp Pathogenic | 15% (1301/8600) European American | 3-5/1000 (9%) | Gochee 2002 Beutler 2002 Gurrin 2009 Pederson 2009 | None reported |

**VARIANT INTERPRETATION:** The His63Asp variant in HFE is a well-studied variant for hereditary hemochromatosis (HH) (Gochee 2002). Although it is considered pathogenic, the penetrance is much reduced. Although 13% of His63Asp homozygous individuals and 17% of Cys282Tyr/His63Asp compound heterozygous individuals exhibit elevated transferrin saturation (Pederson 2009), the clinical penetrance is even lower with ≤5% in individuals who are homozygous or compound heterozygous for pathogenic HFE variants exhibiting symptoms (Beutler 2002, Gurrin 2009). In summary, this variant meets our criteria for pathogenicity but with much reduced penetrance.

**DISEASE INFORMATION:** Hereditary hemochromatosis (HH) is characterized by excessive storage of iron in the liver, skin, pancreas, heart, joints, and testes. In untreated individuals, symptoms may include abdominal pain, weakness, lethargy, weight loss, increased skin pigmentation, diabetes mellitus, congestive heart failure and/or arrhythmias, arthritis, and hypogonadism. The risk of cirrhosis is significantly increased when the serum ferritin is > 1,000 ng/mL. The biochemical and clinical penetrance of HH varies (Beutler 2002, Gurrin 2009, Pederson 2009) and HH is more common in men than women. Adapted from GeneReviews abstract: http://www.ncbi.nlm.nih.gov/books/NBK1440/

**FAMILIAL RISK:** Hereditary hemochromatosis is inherited in an autosomal recessive manner. The risk of this patient's child having the disease is dependent on the carrier status of the patient's partner. Two carriers have a 25% risk for having a child with Hereditary hemochromatosis. Other biologically related family members may also be carriers of this variant.

# PHARMACOGENOMIC ASSOCIATIONS AND BLOOD GROUPS

## C. PHARMACOGENOMIC ASSOCIATIONS

| Drug (Indication) | Summary | Variants Evaluated and Genotypes Identified | Interpretation | References (PMID) |
|---|---|---|---|---|
| Warfarin (Anti-coagulation) | **Standard dose requirement** | *CYP2C9* rs1799853 rs1057910 Genotype: *1/*2 c.[430C;1075A]; c.[430C>T;1075A]<br><br>*VKORC1* rs9923231 Genotype: AA | Patients with the CYP2C9*1/*2 genotype may require a lower dose of warfarin as compared to patients with theCYP2C9*1/*1 genotype. Patients with theVKORC1 AA genotype may require a lower dose of warfarin as compared to patients with the VKORC1 GG or GA genotypes. However, patients with the combination of the CYP2C9*1/*2 genotype and VKORC1 AA genotype are predicted to require standard doses of warfarin compared to other patients. Refer to warfarindosing.org for dosing based on genotype and other clinical factors. | Johnson 2011 |

***VKORC1/CYP2C9* genotype combination frequencies**

| Dosing Group | VKORC1 rs9923231 | CYP2C9 Genotypes | Approximate Frequency |
|---|---|---|---|
| Lower | AA | *1/*3, *2/*2, *2/*3, *3/*3 | 6% |
| | GA | *2/*3, *3/*3 | 3% |
| Standard | AA | *1/*1, *1/*2 | 37% |
| | GA | *1/*2, *1/*3, *2/*2 | 14% |
| | GG | *1/*3, *2/*2, *2/*3 | <1% |
| Higher | GA | *1/*1 | 28% |
| | GG | *1/*1, *1/*2 | 13% |

| Drug (Indication) | Summary | Variants Evaluated and Genotypes Identified | Interpretation | References (PMID) |
|---|---|---|---|---|
| Clopidogrel (Anti-coagulation) | **Typical response to clopidogrel** | *CYP2C19*<br>rs4244285<br>rs4986893<br>rs12248560<br><br>Genotype: *1/*1<br>c.[806C(;)681G(;)636G];[-806C(;)681G(;)636G] | Patients with the CYP2C19 *1/*1 genotype may have extensive (typical) metabolism of clopidogrel as well as well as typical response to clopidogrel as compared to ultrarapid or poor clopidogrel metabolizers. Additional information and dosing recommendations for this result can be found at: http://www.pharmgkb.org/drug/PA449053. | Scott 2013 |

***CYP2C19* genotype frequencies**

| Metabolism | Genotypes | Frequency |
|---|---|---|
| Ultrarapid | *1/*17, *17/*17 | 5-30% |
| Extensive (typical) | *1/*1 | 35-50% |
| Intermediate | *1/*2, *1/*3, *2/17, *3/*17 | 18-35% |
| Poor | *2/*2, *2/*3, *3/*3 | 2-15% |

| Drug (Indication) | Summary | Variants Evaluated and Genotypes Identified | Interpretation | References (PMID) |
|---|---|---|---|---|
| Digoxin (Dysrhythmias, heart failure) | **Intermediate metabolism and serum concentration of digoxin** | *ABCB1*<br>rs1045642<br>Genotype: CT<br><br>*Genotype frequencies:*<br>*CC: 22%* ***CT: 51%*** *TT:27%* | Patients with the CT genotype who take oral digoxin may have intermediate metabolism and serum concentrations of digoxin as compared to patients with the CC and TT genotypes. | Aarnoudse 2008, Kurata 2002, Hoffmeyer 2000 |
| Metformin (Type 2 diabetes mellitus) | **Typical glycemic response to metformin** | C11orf65<br>rs11212617<br>Genotype: TT<br><br>*Genotype frequencies:*<br>***TT:37%*** *TG:48% GG:15%* | Patients with the TT genotype who have Type 2 Diabetes Mellitus and are treated with metformin may have a decreased glycemic response as compared to patients with the GG genotype. An association with increased or decreased glycemic response to metformin was not seen in people diagnosed with impaired glucose tolerance in the absence of Type 2Diabetes Mellitus. | Florez 2012, GoDARTS and UKPDS Diabetes Pharmacogenetics Study Group 2011 |
| Simvastatin (Hyperlipidemia) | **Typical risk of simvastatin-related myopathy** | SLCO1B1<br>rs4149056<br>Genotype: TT<br><br>*Genotype frequencies:*<br>***TT:68%*** *TC:30% CC:2%* | Patients with the TT genotype may have a lower risk of simvastatin-related myopathy as compared to patients with the CT or CC genotype. | Wilke 2012 |

## D. RED BLOOD CELL AND PLATELET ANTIGENS

### SUMMARY

**ABO Rh Blood type: B Negative**

#### Rare RBC Antigens

No rare presence or absence of RBC antigens was identified.

#### Rare Platelet Antigens

No rare presence or absence of platelet antigens was identified.

### DISCUSSION

These red blood cell (RBC) and human platelet antigen (HPA) predictions are based on published genotype to phenotype correlations for the alleles present. Some antigens have also been serologically determined using traditional blood typing methods.
During pregnancy or transfusion alloantibodies to blood group antigens and platelet antigens can form against foreign RBCs that contain immunogenic blood group and platelet antigens that the recipient is missing. These alloantibodies can cause clinically important complications during future transfusions and pregnancy.

#### Blood Production Transfusion

This individual does NOT have an increased risk of forming unusual RBC or platelet alloantibodies, since this test revealed a normal presence of high frequency antigens and no antigen gene rearrangements.

## Blood Production Donation

This individual does NOT pose an increased risk to blood product recipients since this test revealed a normal presence of high frequency antigens and no antigen gene rearrangements.

### RED BLOOD CELL ANTIGENS

| A | B | H | D | C | c | E | e | K | k | Jk(a) | Jk(b) | Fy(a) | Fy(b) |
|---|---|---|---|---|---|---|---|---|---|---|---|---|---|
| - | + | + | - | - | + | - | + | - | + | + | + | + | - |

| M | N | S | S | Lu(a) | Lu(b) | Au(a) | Au(b) | Kp(a) | Kp(b) | Kp(c) | Di(a) | Di(b) |
|---|---|---|---|---|---|---|---|---|---|---|---|---|
| + | - | + | - | [+] | [+] | [+] | [+] | [-] | [+] | [-] | [-] | [+] |

| Wr(a) | Wr(b) | Yt(a) | Yt(b) | Sc1 | Sc2 | Do(a) | Do(b) | Jo(a) | Hy | Co(a) | Co(b) | LW(a) | LW(b) |
|---|---|---|---|---|---|---|---|---|---|---|---|---|---|
| [-] | [+] | [+] | [-] | [+] | [-] | [+] | [-] | [+] | [+] | [+] | [-] | [+] | [-] |

| Cr(a) | Kn(a) | Kn(b) | Sl(a) | Vil | Yk(a) | KCAM | McC(a) | McC(b) | In(a) | In(b) |
|---|---|---|---|---|---|---|---|---|---|---|
| [+] | [+] | [-] | [+] | [-] | [+] | [+] | [+] | [-] | [-] | [+] |

| Ok(a) | MER2 | JMHK | JMHL | FORS |
|---|---|---|---|---|
| [+] | [+] | [+] | [+] | [-] |

### PLATELET ANTIGENS

| 1a | 1b | 2a | 2b | 3a | 3b | 4a | 4b | 5a | 5b | 6bw | 7bw | 8bw | 9bw |
|---|---|---|---|---|---|---|---|---|---|---|---|---|---|
| [+] | [+] | [+] | [-] | [+] | [-] | [+] | [-] | [+] | [-] | [-] | [-] | [-] | [-] |

| 10bw | 11bw | 12bw | 13bw | 14bw | 15a | 15b | 16bw | 17bw | 18bw | 19bw | 20bw | 21bw | 22bw |
|---|---|---|---|---|---|---|---|---|---|---|---|---|---|
| [-] | [-] | [-] | [-] | [-] | [+] | [+] | [-] | [-] | [-] | [-] | [-] | [-] | [-] |

| 23bw | 24bw | 25bw | 26bw | 27bw |
|---|---|---|---|---|
| [-] | [-] | [-] | [-] | [-] |

Key: [+] presence of antigen predicted by genotyping; + presence of antigen predicted by genotyping and confirmed by serology; +* presence of antigen detected by serology, genotype prediction not available; [+w] weak presence of antigen predicted by genotyping; +w weak presence of antigen predicted by genotyping and confirmed by serology; +w* weak presence of antigen detected by serology, genotype prediction not available; [−] absence of antigen predicted by genotyping; - absence of antigen predicted by genotyping and confirmed by serology, -* absence of antigen detected by serology, genotype prediction not available; NC indicates no sequencing coverage, Dis indicates discordant. Rare (less than 5% population frequency) presence or absence of antigen is indicated in **red**.

## METHODOLOGY

Genomic sequencing is performed using next generation sequencing on the Illumina HiSeq platform. Genomes are sequenced to at least 30X mean coverage and a minimum of 95% of bases are sequenced to at least 8X coverage. Paired-end 100bp reads are aligned to the NCBI reference sequence (GRCh37) using the Burrows-Wheeler Aligner (BWA), and variant calls are made using the Genomic Analysis Tool Kit (GATK). Variants are subsequently filtered to identify: (1) variants classified as disease causing in public databases; (2) nonsense, frameshift, and +/-1,2 splice-site variants that are novel or have a minor allele frequency <1% in European American or African American chromosomes from the NHLBI Exome Sequencing Project (http://evs.gs.washington.edu/EVS/); and (3) rs11212617 (C11orf65; metformin), rs12248560 (CYP2C19; clopidogrel), rs4244285 (CYP2C19; clopidogrel), rs4986893 (CYP2C19; clopidogrel), rs28399504 (CYP2C19; clopidogrel), rs41291556 (CYP2C19; clopidogrel), rs72552267 (CYP2C19; clopidogrel), rs72558186 (CYP2C19; clopidogrel), rs56337013 (CYP2C19; clopidogrel), rs1057910 (CYP2C9; warfarin), rs1799853 (CYP2C9; warfarin), rs7900194 (CYP2C9; warfarin), rs9332131 (CYP2C9; warfarin), rs28371685 (CYP2C9; warfarin), rs28371686 (CYP2C9; warfarin), rs9923231 (VKORC1; warfarin), rs4149056 (SLCO1B1; simvastatin), and rs1045642 (ABCB1; digoxin). The evidence for phenotype-causality is then evaluated for each variant resulting from the filtering strategies above and variants are classified according to LMM criteria (http://pcpgm.partners.org/LMM). Only those variants with evidence for causing highly penetrant disease or contributing to disease in a recessive manner are reported. Before reporting, all variants are confirmed via Sanger sequencing or another orthogonal technology. The initial sequencing component of this test was performed by the Illumina Clinical Services Laboratory (San Diego, CA CLIA# 05D1092911) and the alignment, variant calling, data filtering, Sanger confirmation and interpretation were performed by the Laboratory for Molecular Medicine at the Partners Healthcare Center for Personalized Genetic Medicine (Cambridge, MA

CLIA#22D1005307). This test has not been cleared or approved U.S. Food and Drug Administration (FDA). The FDA has determined that such clearance or approval is not necessary.

## LIMITATIONS

It should be noted that this test does not sequence all bases in a human genome and not all variants have been identified or interpreted. Triplet repeat expansions, translocations and large copy number events are currently not reliably detected by genome sequencing. Furthermore, not all disease-associated genes have been identified and the clinical significance of variation in many genes is not well understood. It is recommended that genomic sequencing data is periodically reinterpreted, especially when new symptoms arise.

## REFERENCES

Aarnoudse AJ, Dieleman JP, Visser LE, Arp PP, van der Heiden IP, van Schaik RH, Molokhia M, Hofman A, Uitterlinden AG, Stricker BH. 2008. Common ATP-binding cassette B1 variants are associated with increased digoxin serum concentration. Pharmacogenet. Genomics. 18(4):299-305.

Beutler E, Felitti VJ, Koziol JA, Ho NJ, Gelbart T. 2002. Penetrance of 845G--> A (C282Y) HFE hereditary haemochromatosis mutation in the USA. Lancet. 359(9302):211-8.

Florez JC, Jablonski KA, Taylor A, Mather K, Horton E, White NH, Barrett-Connor E, Knowler WC, Shuldiner AR, Pollin TI, Diabetes Prevention Program Research Group. 2012. The C allele of ATM rs11212617 does not associate with metformin response in the Diabetes Prevention Program. Diabetes Care. 35(9):1864-7.

Gochee PA, Powell LW, Cullen DJ, Du Sart D, Rossi E, Olynyk JK. 2002. A population-based study of the biochemical and clinical expression of the H63D hemochromatosis mutation. Gastroenterology. 122(3):646-51.

GoDARTS and UKPDS Diabetes Pharmacogenetics Study Group, Wellcome Trust Case Control Consortium 2, Zhou K, Bellenguez C, Spencer CC, Bennett AJ, Coleman RL, Tavendale R, Hawley SA, Donnelly LA, Schofield C, Groves CJ, Burch L, Carr F, Strange A, Freeman C, Blackwell JM, Bramon E, Brown MA, Casas JP, Corvin A, Craddock N, Deloukas P, Dronov S, Duncanson A, Edkins S, Gray E, Hunt S, Jankowski J, Langford C, Markus HS, Mathew CG, Plomin R, Rautanen A, Sawcer SJ, Samani NJ, Trembath R, Viswanathan AC, Wood NW, MAGIC investigators, Harries LW, Hattersley AT, Doney AS, Colhoun H, Morris AD, Sutherland C, Hardie DG, Peltonen L, McCarthy MI, Holman RR, Palmer CN, Donnelly P, Pearson ER. 2011. Common variants near ATM are associated with glycemic response to metformin in type 2 diabetes. Nat. Genet. 43(2):117-20.

Gurrin LC, Bertalli NA, Dalton GW, Osborne NJ, Constantine CC, McLaren CE, English DR, Gertig DM, Delatycki MB, Nicoll AJ, Southey MC, Hopper JL, Giles GG, Anderson GJ, Olynyk JK, Powell LW, Allen KJ, HealthIron Study Investigators. 2009. HFE C282Y/H63D compound heterozygotes are at low risk of hemochromatosis-related morbidity. Hepatology. 50(1):94-101.

Hoffmeyer S, Burk O, von Richter O, Arnold HP, Brockmöller J, Johne A, Cascorbi I, Gerloff T, Roots I, Eichelbaum M, Brinkmann U. 2000. Functional polymorphisms of the human multidrug-resistance gene: multiple sequence variations and correlation of one allele with P-glycoprotein expression and activity in vivo. Proc. Natl. Acad. Sci. U.S.A. 97(7):3473-8.

Johnson JA, Gong L, Whirl-Carrillo M, Gage BF, Scott SA, Stein CM, Anderson JL, Kimmel SE, Lee MT, Pirmohamed M, Wadelius M, Klein TE, Altman RB, Clinical Pharmacogenetics Implementation Consortium. 2011. Clinical Pharmacogenetics Implementation Consortium Guidelines for CYP2C9 and VKORC1 genotypes and warfarin dosing. Clin. Pharmacol. Ther. 90(4):625-9.

Kurata Y, Ieiri I, Kimura M, Morita T, Irie S, Urae A, Ohdo S, Ohtani H, Sawada Y, Higuchi S, Otsubo K. 2002. Role of human MDR1 gene polymorphism in bioavailability and interaction of digoxin, a substrate of P-glycoprotein. Clin. Pharmacol. Ther. 72(2):209-19.

Kujovich JL. 2011. Facor V Leiden thrombophilia. Genet. Med. 13(1):1-16.

Pedersen P, Milman N. 2009. Genetic screening for HFE hemochromatosis in 6,020 Danish men: penetrance of C282Y, H63D, and S65C variants. Ann. Hematol. 88(8):775-84.

Scott SA, Sangkuhl K, Stein CM, Hulot JS, Mega JL, Roden DM, Klein TE, Sabatine MS, Johnson JA, Shuldiner AR, Clinical Pharmacogenetics Implementation Consortium. 2013. Clinical Pharmacogenetics Implementation Consortium guidelines for CYP2C19 genotype and clopidogrel therapy: 2013 update. Clin. Pharmacol. Ther. 94(3):317-23.

Simone B, De Stefano V, Leoncini E, Zacho J, Martinelli I, Emmerich J, Rossi E, Folsom AR, Almawi WY, Scarabin PY, den Heijer M, Cushman M, Penco S, Vaya A, Angchaisuksiri P, Okumus G, Gemmati D, Cima S, Akar N, Oguzulgen KI, Ducros V, Lichy C, Fernandez-Miranda C, Szczeklik A, Nieto JA, Torres JD, Le Cam-Duchez V, Ivanov P, Cantu-Brito C, Shmeleva VM, Stegnar M, Ogunyemi D, Eid SS, Nicolotti N, De Feo E, Ricciardi W, Boccia S. 2013. Risk of venous thromboembolism associated with single and combined effects of Factor V Leiden, Prothrombin 20210A and Methylenetethraydrofolate reductase C677T: a meta-analysis involving over 11,000 cases and 21,000 controls. Eur. J. Epidemiol. 28(8):621-47.

**GENOME REPORT (CONTINUED)**

Wilke RA, Ramsey LB, Johnson SG, Maxwell WD, McLeod HL, Voora D, Krauss RM, Roden DM, Feng Q, Cooper-Dehoff RM, Gong L, Klein TE, Wadelius M, Niemi M, Clinical Pharmacogenomics Implementation Consortium (CPIC). 2012. The clinical pharmacogenomics implementation consortium: CPIC guideline for SLCO1B1 and simvastatin-induced myopathy. Clin. Pharmacol. Ther. 92(1):112-7.

Wissinger B, Gamer D, Jägle H, Giorda R, Marx T, Mayer S, Tippmann S, Broghammer M, Jurklies B, Rosenberg T, Jacobson SG, Sener EC, Tatlipinar S, Hoyng CB, Castellan C, Bitoun P, Andreasson S, Rudolph G, Kellner U, Lorenz B, Wolff G, Verellen-Dumoulin C, Schwartz M, Cremers FP, Apfelstedt-Sylla E, Zrenner E, Salati R, Sharpe LT, Kohl S. 2001. CNGA3 mutations in hereditary cone photoreceptor disorders. Am. J. Hum. Genet. 69(4):722-37.
